# Algorithm-supported, mass and sequence diversity-oriented random peptide library design

**DOI:** 10.1186/s13321-019-0347-6

**Published:** 2019-03-28

**Authors:** Daniela Kalafatovic, Goran Mauša, Toni Todorovski, Ernest Giralt

**Affiliations:** 1 Institute for Research in Biomedicine (IRB Barcelona), The Barcelona Institute of Science and Technology (BIST), Baldiri Reixac 10, 08028 Barcelona, Spain; 2 Faculty of Engineering, University of Rijeka, Vukovarska 58, 51000 Rijeka, Croatia; 3 Department of Inorganic and Organic Chemistry, University of Barcelona, Marti i Franques 1-5, 08028 Barcelona, Spain

**Keywords:** Peptide libraries, One-bead-one-compound, Algorithm-supported design, Genetic algorithm, Optimization

## Abstract

Random peptide libraries that cover large search spaces are often used for the discovery of new binders, even when the target is unknown. To ensure an accurate population representation, there is a tendency to use large libraries. However, parameters such as the synthesis scale, the number of library members, the sequence deconvolution and peptide structure elucidation, are challenging when increasing the library size. To tackle these challenges, we propose an algorithm-supported approach to peptide library design based on molecular mass and amino acid diversity. The aim is to simplify the tedious permutation identification in complex mixtures, when mass spectrometry is used, by avoiding mass redundancy. For this purpose, we applied multi (two- and three-)-objective genetic algorithms to discriminate between library members based on defined parameters. The optimizations led to diverse random libraries by maximizing the number of amino acid permutations and minimizing the mass and/or sequence overlapping. The algorithm-suggested designs offer to the user a choice of appropriate compromise solutions depending on the experimental needs. This implies that diversity rather than library size is the key element when designing peptide libraries for the discovery of potential novel biologically active peptides.

**Electronic supplementary material:**

The online version of this article (10.1186/s13321-019-0347-6) contains supplementary material, which is available to authorized users.

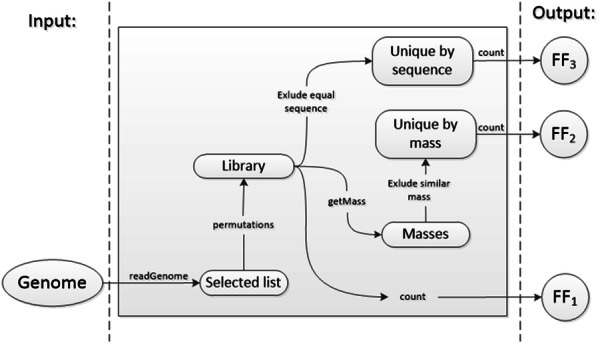

## Introduction

Small-molecule libraries are widely used in drug discovery to identify biologically active molecules [1]. Traditionally, small molecule library design is based on a known target structure or on known ligands. The last two decades have witnessed the application of docking calculations to the pharmaceutical field through the lead optimization of structure-based design of small molecules [2]. Moreover, molecular docking is the main approach in the structure-based peptidyl-drug design studies, even though peptide based drugs are less explored than the small molecule ones. However, when the targets and/or ligands are unknown or “undruggable” this virtual screening approach fails to provide useful information [3]. Therefore, one has to rely on experimental screenings of random, large numbers of compounds in order to obtain further insight into possible identification of binders or disruptors of protein-protein interactions (PPI) [4, 5].

Safer and more specific alternatives to small molecules, peptide-based drugs are emerging as a new paradigm in medicinal chemistry [6, 7]. Therefore, there is a need to develop combinatorial approaches to identify new peptide therapeutics [5]. In this context, phage display was the main approach to obtain a variety of random peptide sequences [8, 9]. Nowadays, the phage display technology is well established in the peptide-based drug discovery process [10, 11]. However, the limitation was that the library building blocks are limited to the 20 natural proteinogenic amino acids resulting in peptides with short half-lives and susceptible to proteolytic cleavage. Progress with phage display and RNA-display technologies has allowed the use of non-proteinogenic amino acids in those techniques [12], but the one-bead-one-compound (OBOC) method allows a more straightforward use of unnatural amino acids [13, 14]. It allows the introduction of stereochemical variability at the $\alpha$-carbon, head-to-tail cyclization, disulfide bridges, etc. and their combinations without complex genetic manipulations [15]. Therefore, OBOC peptide libraries, have opened up the path to a larger search space for discovery of peptide-based drugs [15, 16].

Chemical and molecular diversity of library components are key for drug discovery [17]. Combinatorial libraries are tools that allow large amount of compounds to be screened at the same time [4]. Currently, the diversity-oriented systems (DOS) for small molecules are taking over the static combinatorial approach, allowing the introduction of skeletal, structural and stereo-chemical complexity [18–20]. Similarly to DOS, one can think of introducing stereo-chemical and skeletal complexity into peptides through the use of D-stereoisomers, the combination of L- and D-ones, the retro-enantio versions and cyclization. By this means, peptide-based libraries with increased stability could be generated. However, their analysis is challenging as similarity in structure and behavior of available peptide permutations would lead to the impossibility to distinguish between sequences using today’s available screening and/or analyzing techniques such as HPLC, UPLC-MS, etc.

It is believed that larger the sample size, more accurate is the representation of a given population [21]. In these terms, random libraries are advantageous over the focused ones, as they cover a larger search space. However, parameters such as the synthesis scale to guarantee the synthesis of all possible compounds, the number of library members, the sequence deconvolution and peptide structure elucidation after screening, are challenging steps when increasing the library size [22]. Moreover, libraries may result in competition among candidates and biased hits identification due to the physico-chemical similarity of the components [21]. This is particularly the case during the sequence deconvolution process of positive hits, where the identification of single peptides is not always possible. Often, families of peptides having identical masses are identified [23]. In order to address this issue, we set out to determine whether multi-objective genetic algorithms can aid and simplify the rational design of random libraries to increase diversity and minimize redundancy. The advantage of this tool is the reduction of the number of library members in a single screening while maintaining maximal chemical and mass diversity.

**Scheme 1:**
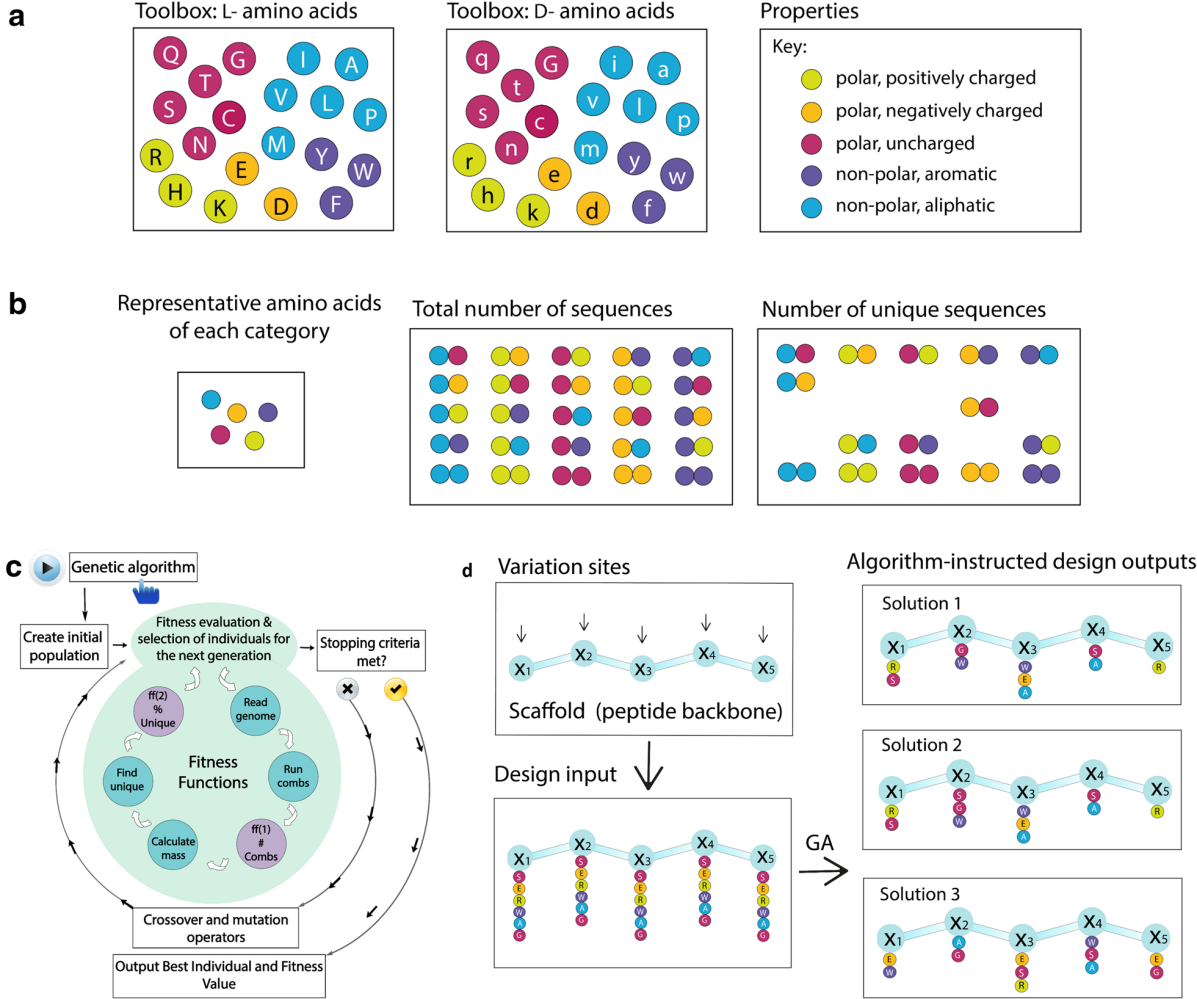
Towards the algorithm-supported design of random peptide libraries. **a** Amino acid toolbox of L- and D-stereoisomers of 20 proteinogenic amino acids color-coded to represent a specific property: hydrophobic-aromatic (magenta), hydrophobic-aliphatic (blue), hydrophilic-uncharged (purple), hydrophilic-positively charged (green), and hydrophilic-negatively charged (orange). **b** Graphical representation of building an OBOC peptide library, based on dipeptides (r = 2), using the representative amino acids from the toolbox (m = 5), depicting the advantage of working with sequences having unique masses. The calculator explores the full sequence space ($5^2=25$) and excludes permutations that present the same mass and differ only in the amino acid order within the sequence. **c** Schematic representation of the 2-objective genetic algorithm used to perform the optimization, indicating the steps required to perform the selection of individuals for the next generation. The main criteria of the GA are two fitness functions for maximizing (1) the number of all amino acid permutations and (2) the number of sequences having unique mass. **d** Schematic representation of the optimization algorithm input and outputs suggesting the best design options (for each position *r*) to obtain maximally diverse random peptide libraries

Recently, artificial intelligence has been applied to a variety of chemical problems to maximize the chance of successful and rapid solving of complex issues [24–28]. In our group, evolutionary algorithms have been successfully applied to address various challenges of peptide chemistry [29–32] related to the design and identification of peptides that cross the blood-brain-barrier (BBB) [33], or that bind to the major histocompatibility complex (MHC) [34]. Recently, Cronin and co-workers used evolutionary algorithms in combination to machine learning to predict antimicrobial activity of peptide sequences [35]. Although the heuristic approach has been explored in the field of peptide design, it is mainly focused on the use of the one-objective setting where one fitness function optimizes a specific peptide property (e.g., antimicrobial, anticancer activity) [36–38]. To date, multi-objective genetic algorithms have been applied to the design of small molecule combinatorial libraries [21, 39–41] but have not been explored for random peptide libraries yet. Herein, we present the framework to solving and/or simplifying a combinatorial challenge—whether we could rationally design a random peptide library and what are the rules that underline this algorithm supported process. For this purpose, we used an evolutionary computing approach based on a multi-objective genetic algorithm (GA) [42, 43] represented in scheme 1 to address the issue of maximizing the number of diverse amino acid permutations. In this way, sequence deconvolution and peptide sequence elucidation are simplified by avoiding sequence redundancy. Ultimately, the designer is able to make an algorithm-supported choice of an appropriate compromise solution among given optimized library designs, depending on the experimental needs.

## Results and discussion

The 20 gene-encoded amino acids, together with a variety of non-natural ones, constitute a versatile toolbox for combinatorial library design (Scheme 1a). A high chemical and structural diversity can be achieved by designing libraries composed of sequences of amino acids (peptides) which total number is calculated using the formula $R=m^r$, where *r* is the number of positions where the variability can be introduced and m the number of amino acids per position [22]. Therefore, the number of permutations grows exponentially with the peptide length, depending also on the number of amino acids per position. If all the 20 proteinogenic amino acids are used in *r* positions, there are $R=20^r$ possible permutations. OBOC libraries prepared using the “split and mix” methodology result in redundant peptide mixtures consisting of permutations of amino acids having overlapping masses [23].

In our study, the introduction of complexity of the system refers to the molecular weight ($M_W$) diversity of the library components. In the case of peptides, made of combinations of various natural or non-natural amino acids, the $M_W$ diversity often corresponds to sequence diversity. Therefore, based on monoisotopic $M_W$ values we set out to determine an algorithm supported approach to help the random design of systems that maintain maximum mass and sequence diversity. Ultimately, the objective of this paper is to simplify the tedious and sometimes difficult chromatographic and mass spectrometry based analyses of complex mixtures of peptides that have similar properties. We chose the $M_W$ as the discriminant for increasing diversity and complexity of the system, consequently reducing the number of peptides in the mixture.

**Fig. 1:**
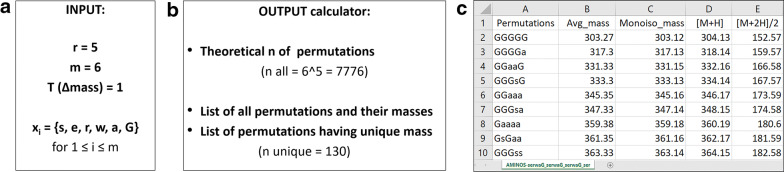
Peptide calculator inputs and outputs. **a** Summary of the input to the peptide mass calculator indicating the number of positions (r = 5) where variability can be introduced and the possible amino acids for each position (m = 6; being $x_i={s,e,r,w,a,G}$) and $T(\Delta mass)=1$. **b** List of outputs of the peptide mass calculator showing the theoretical number of possible permutations (= 7776) and the number of permutations having unique masses (= 130) alongside their list. **c** A screenshot of a part of the output file in the csv format

First, we developed a peptide mass calculator with the possibility to discriminate between masses of peptides in a given mixture, generating an output csv (comma-separated values) file containing all possible peptide permutations alongside with their masses in addition to a csv file containing the list of peptides with unique masses. In the latter, the permutations having the same amino acid composition but with varying locations within the sequence were omitted except one representative of each collision set. The same was applied to peptides having different amino acid composition but overlapping masses, with tolerance set to 1 (see Additional file 1: Fig. S7). Tolerance is an arbitrary parameter that is user defined and it can be tuned to any value, as described in the next subsection. A simplified schematic representation of the calculator output, using a small library of dipeptides is shown in Scheme 1b. The amino acids are divided in 5 different categories (color-coded) depending on their polarity and charge (Scheme 1a). If each of the colors stands for one representative amino acid and all the possible dipeptides are made, there are $5^2$ (r = 2, m = 5) = 25 permutations. After the calculator excludes the peptides with overlapping masses, we are left with a subset of 15 dipeptides of unique masses, as shown in Scheme 1b (right panel). In this way, we could simplify the chromatographic and mass spectrometry analysis of the mixture.

The following step was the development of the optimization tool based on the genetic algorithm (Scheme 1c) that is able to suggest several design strategies based on user’s needs and inputs (Scheme 1d). The input is the same as for the calculator, where the number of positions (*r*) and the amino acids ($x_i$) for each position have to be defined. The output is a Pareto front containing a distribution of solutions, based on how they scored during the GA selection rounds. The Pareto front is a set of non-dominated solutions chosen as optimal by the GA. Subsequently, the user makes the choice of the preferred design suggestion based on the experimental expertise and the requirements of the library (Scheme 1d). We refer to this choice as algorithm-supported decision. Moreover, we explored the possibility of a three objective algorithm to include also the sequence diversity.

### **Library mass analysis with the combinatorial calculator**

During the initial step of peptide library planning there are two parameters that need to be determined (Fig. 1a): (1) the number of positions *r* and (2) the list of $m_i$ amino acids $x_i=\left\{ \right\}$ that can appear at *i*-th positions, where $1 \le i \le r$. These constitute our input parameters. To simplify and automatize mass calculations of all the possible permutations in a user-defined peptide library, the peptide mass calculator algorithm was developed using the Matlab scripting language. An example is a pentapeptide library where the input was: r = 5, m = 6, for $x_i$ = s, e, r, w, a, G (Fig. 1a). For this condition, the total number of peptide permutations given by $m^r$ is 7776 (Fig. 1b).

The peptide mass calculator also compares the masses of all the possible permutations and locates the ones that have unique mass. The motivation behind this was to estimate whether the analysis of all the compounds with unique masses i.e., simplified libraries in terms of number of peptides in the mixture is feasible by using chromatography coupled mass spectrometry. Hence, the mass of a peptide M is considered unique if no other peptide has the mass within the range $<M - T(\Delta mass), M + T(\Delta mass)>$. If there is another peptide in the mixture, having mass within this range, it is excluded from the list of permutations unique by mass, thus automatizing redundancy identification. The parameter $T(\Delta mass)$ is referred to as the tolerance of mass discrimination. It is also a user-defined parameter, which may be tuned (according to user’s needs and the resolution of the machine used to perform the mass spectrometry). Throughout this study, the condition $T(\Delta mass)=1$ was kept in all the calculations and permutations, unless stated otherwise. Using this configuration and the exemplary pentapeptide library from Fig. 1a, the peptide mass calculator computes 130 peptides of unique mass (Fig. 1b).

The calculator output provides a csv (comma-separated value) type file containing the full list of permutations with the corresponding $M_W$ values, in the form of: (1) the average mass, (2) the monoisotopic mass, (3) the singly charged $[M+H]^+$ and (4) the doubly charged $[M+2H]^{2+}$ expected ions for mass spectrometry analysis (Fig. 1c). The algorithm also provides the full list of unique peptide masses (for simplicity only a screenshot is shown in the Fig. 1c).

**Fig. 2:**
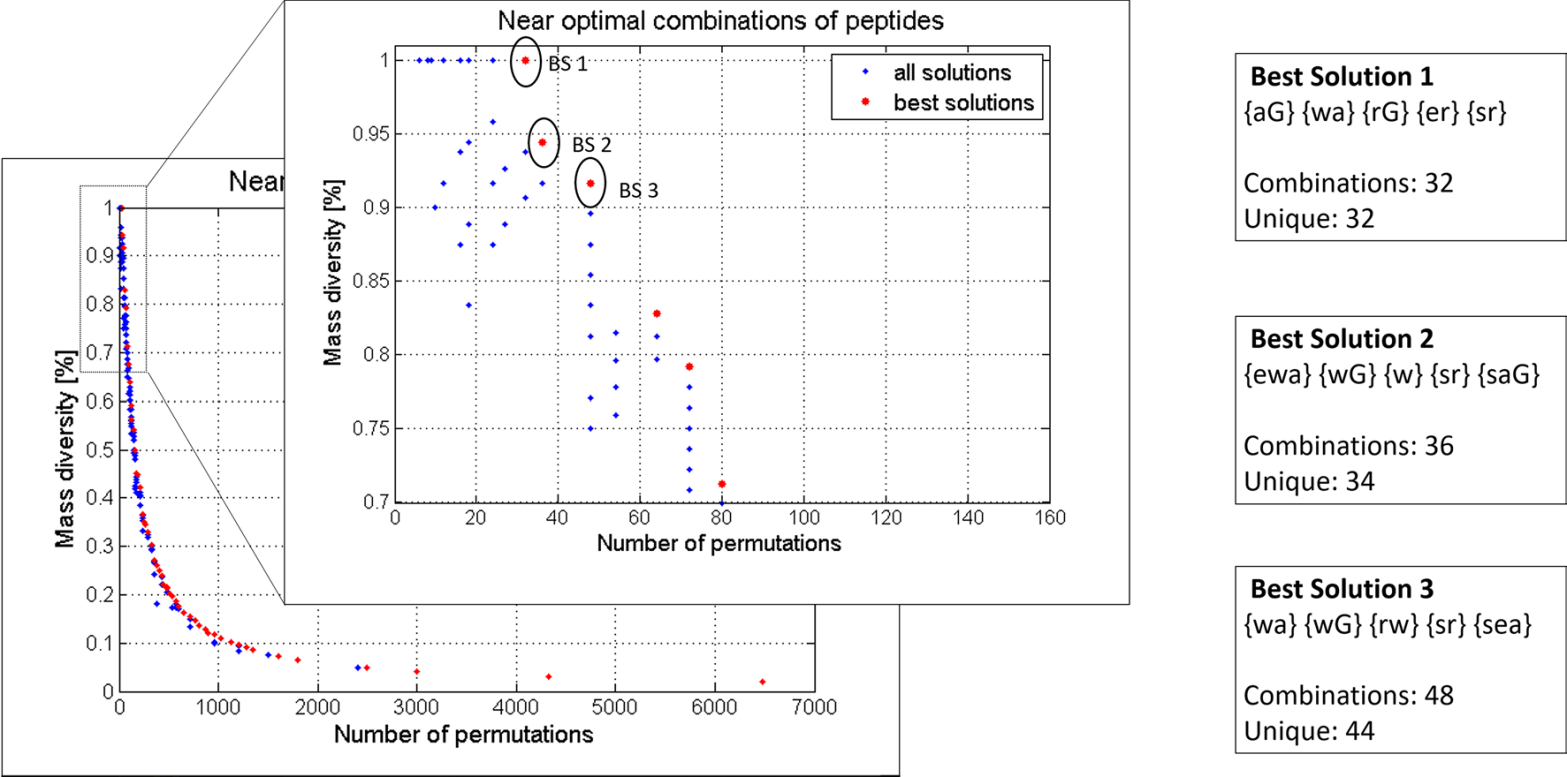
Genetic algorithm-informed design suggestions. Output of the optimization genetic algorithm used to identify the best library designs, showing the pareto front (and the zoom of the 70–100% variability region of the pareto front) with all the working solutions (blue) and the best solutions (red) from the final population, obtained using the same input as in Fig. 1a. Three representative solutions (BS 1, BS 2 and BS 3 corresponding to 100%, 94% and 92% respectively) have been selected as suggestions for three possible designs to obtain “simplified” and maximally diverse OBOC libraries

Therefore, our algorithm gives the possibility to the user to explore all possible permutations as well as the ones baring unique masses, representative of the population. Here, peptide diversity is achieved solely through the introduction of functional group (side chain) diversity and leads to peptide collections with variable mass diversity. This tool is the basis for the optimization process used for the algorithm-supported library design described below.

### **Library design optimization with the genetic algorithm approach**

Next, we developed an optimization decision support system based on the genetic algorithm (GA) approach. GAs are iterative heuristic processes of computational optimization that is based on selection, cross-over and mutation of genes, analogous to the natural process of evolution where the best genes of individuals are preserved and passed to the next generation with the expectation that the fittest parents will give even fitter offspring. In computer science, GAs are mimicking this process by trial and error, gradually finding the best solution or solutions to a given problem and ending only if one of the stopping criteria is met (Fig. 1c): (1) the fitness function of an individual achieved the maximum level or a predefined threshold level, i.e., the algorithm has found the best solution or a solution that is good enough; (2) after a certain number of generations, the fitness function of best solutions is not improved, i.e., the algorithm cannot find another solution that is better than the one it has already found. In each iteration (generation) of a GA, a group (population) of candidate solutions (individuals) to a given optimization problem are considered simultaneously. Each solution is defined by parameters encoded in genes and its quality is quantified by fitness functions. The values of fitness functions are numerical indicators used to compare solutions and find the best ones.

**Fig. 3:**
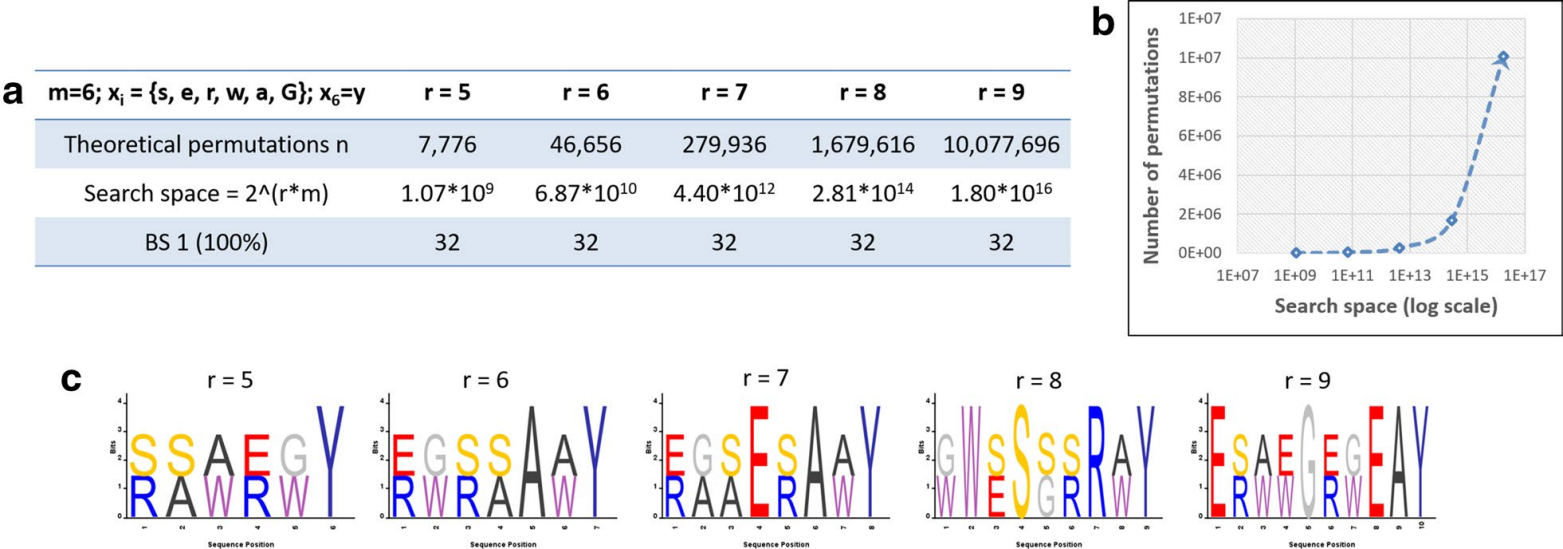
Optimization of an exemplary multi-peptide library (T($\Delta$ mass)=1). **a** Input for the optimization genetic algorithm indicating the varying number of positions *r*, the number of theoretical permutations *n*, the size of search space, and the number of peptides in the BS 1 for each *r*. **b** The rate of change of the search space, presented in logarithmic scale (x-axis) in comparison to the exponential growth of the total number of permutation (y-axis). **c** Sequence logos of BS 1, where the percentage of peptides diverse by mass is 100%

In this paper, the second variant of the Non-dominated Solutions Genetic Algorithm (NSGA-II) was used to run peptide library optimizations. The goal was to identify optimal library designs that yield the greatest number of peptides with unique masses. The motivation was to find library designs with feasible analysis of all its components by chromatography coupled mass spectrometry. The design of a library is defined by the number of positions r and the variability (a number of possible amino acids) in each position $x_i$ $(1< i < r)$. The variability is user defined and consists of a set of possible amino acids, which may vary for each position. The implemented algorithm transforms each set of possible amino acids into a bit-string, where the length of the bit-string is equal to the length of the set and each bit (1 or 0) represents the inclusion (1) or exclusion (0) of a given amino acid. That way, the algorithm is searching for an optimal subset of amino acids. The search is guided by two fitness functions that need to be maximized: (1) the total number of peptides for a given variability and (2) the number of peptides unique by mass. Both fitness functions are computed for each solution examined in this iterative search process by using the peptide mass calculator.

To illustrate the advantage of the optimization output over the single, user-predetermined design, we used the same input shown in Fig. 1a. In contrast to the peptide calculator, the optimization offers a wide range of random peptide library designs simplified to satisfy the maximum mass diversity condition. This can be seen from the Pareto front that contains all the best solutions (red dots) and the distribution of all the remaining solutions from the final population (blue dots) in Fig. 2. We focused our analysis to the 70% to 100% diversity region and reported on the three best solutions encircled in the zoomed Pareto front graph being BS 1 (100%), BS 2 (94%) and BS 3 (92%). Our implementation of the algorithm also allows the user to pick any point of interest and obtain a detailed analysis containing the output of the peptide mass calculator accompanied by the corresponding sequence logo graphs. All the sequence logos throughout the text are presented in the conventional N- to C-terminus fashion.

**Fig. 4:**
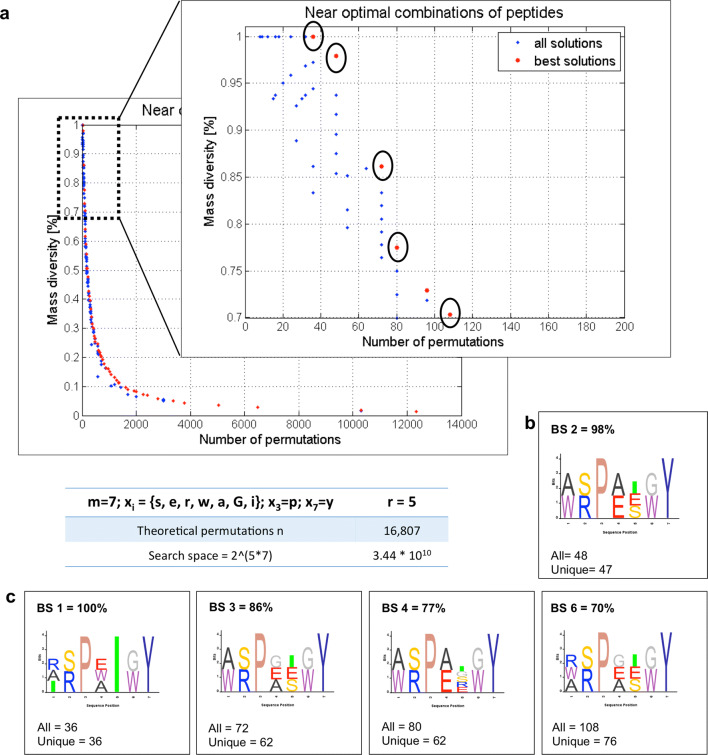
Two-objective genetic algorithm-informed design suggestions for experimental validation. **a** Pareto front (output) of the optimization results for the OBOC peptide library having 5 positions where variability was introduced (r = 5), with m = 7 and $x_i={s,e,r,w,a,G,i}$ and two fixed positions, being $x_3=p$ and $x_7=y$. In the zoom of the pareto front, in the 70–100% mass diversity range, we chose five best solutions: BS 1 (100%), BS 2 (98%), BS 3 (86%), BS 4 (77%) and BS 6 (70%). **b** Sequence logo representation of the BS 2, showing the maximally diverse random peptide library design we would choose for further studies. **c** Sequence logos of BS 1, BS 3, BS 4 and BS 6 suggesting various synthetic possibilities and pointing out possible synthetic challenges. Several other design suggestions are available, but we show only these five for simplicity

The library design suggestion for BS 1 consists of 32 peptides having unique mass. The algorithm informs that this specific library can be obtained by simplifying the input from *s, e, r, w, a, G* in each position x_i_ to {*a, G*} for $x_1$, {*w, a*} for $x_2$, {*r, G*} for $x_3$, {*e, r*} for $x_4$ and {*s, r*} for $x_5$ (Fig. 2). Similar design suggestions are shown in Fig. 2 for BS 2 and BS 3, where the number of peptides having unique masses increases as well as the mass overlapping. Depending on users requirements, in terms of the number of library members and the desired chemical diversity, the design strategy can shift towards the increasing number of possible peptide permutations and introduction of limited or extended mass overlapping. From the same optimization, BS 50%, BS 30% and BS 10% points were also analyzed. The zoom of the Pareto front in the 10% to 50% mass diversity range with the corresponding sequence logos can be found in supplementary information (Additional file 1: Fig. S1). Having increased the number of amino acids in each position $x_{i}$ lead to the greater number of peptides unique by mass in BS 50%, BS 30% and BS 10%. However, the total number of peptides increased at a greater rate, thus reducing the overall mass diversity ratio of these libraries.

The size of search space is the number of all possible solutions for the binary encoded genome calculated as $2^{(r * m)}$ (Fig. 3a). In our case, the complexity of the optimization task is determined by the search space size that is exponentially dependent on the product of *m* and *r*. In Fig. 3, we showed how the search space changes from $10^9$ to $10^{16}$ for a constant value of *m* ($m=6$) when increasing *r* from 5 to 10, respectively. The time needed to complete the optimization task is influenced by its complexity (i.e. balance of *m* and *r*) and the available computer resources. For peptide lengths from 5 to 10-residues long with varying values of *m* the algorithm completed the optimization tasks in a reasonable time frame (hours to days) with a standard PC configuration. In contrast to the search space complexity for the given set of libraries (Fig. 3b), all the best solutions (BS 1) have only 32 peptides unique by mass, regardless of the increasing number of positions *r* (Fig. 3a). A set of sequence logo graphs given in Fig. 3c corresponding to 100% mass diversity shows a set of designs available to the user. Their similarity proves that having an algorithm-supported design strategy still requires carefully chosen inputs from the expert user.

To illustrate the utility of the two-objective optimization, we used the example of an all D-heptapeptide with r = 5 and m = 7, for $x_i = {s, e, r, w, a, G, i}$; having two fixed positions $x_3=p$ and $x_7=y$. The Pareto front for this optimization can be seen in Fig. 4a. When zoomed into the 70–100% mass diversity region, several design solutions appear. We chose to analyze five points being BS 1 (100%), BS 2 (98%), BS 3 (86%), BS 4 (77%) and BS 6 (70%) and show their sequence logos (Fig. 4b and c) to allow better visualization of the design suggestions and possible synthetic challenges. In our region of interest, the algorithm suggests possible designs to obtain maximally diverse random peptide libraries. Interestingly, BS 2 shows a high mass diversity of 98% and only one peptide with overlapping mass. With 47 peptides unique by mass it offers a design possibility with 33% higher total number of peptides and only 2% mass diversity reduction when compared to BS 1. Thus, this would be our design of choice to attempt the experimental validation. The complete list of peptides for this solution can be found in the Additional file 2: Table S1.

To show the versatility of the optimization process, we presented several other examples of library designs: (a) r = 5, m = 6 for $x_i={s, e, r, w, a, G}$; $x_6=y$ in Additional file 1: Fig. S2, (b) r = 7, m = 7 for $x_i={w, r, e, p, s, i, a}$; $x_7=y$ in Additional file 1: Fig. S3, (c) r = 5, m = 10 for $x_i={h, f, r, a, n, e, s, y, w, i}$ in Additional file 1: Fig. S4 and (d) r = 6, m = 10 for $x_i={h, f, r, a, n, e, s, y, w, i}$; $x_6=G$ in Additional file 1: Fig. S5.

**Fig. 5:**
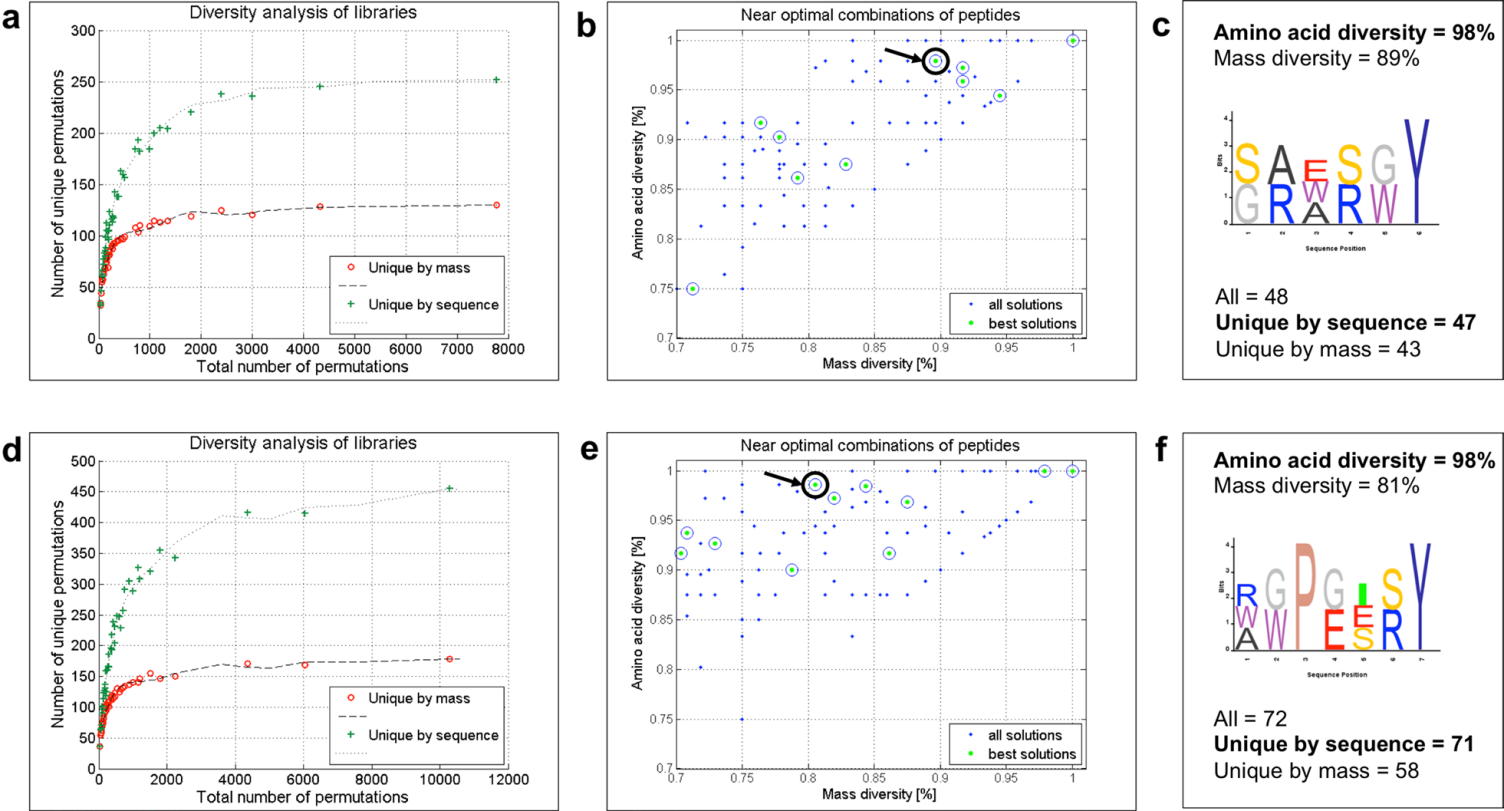
Three-objective genetic algorithm-informed design suggestions. **a** Diversity analysis of all the best solutions obtained by the 3-objective optimization for (r = 5, m = 6 for $x_i={s, e, r, w, a, G}$; $x_6=y$) library design in terms of the number of unique permutations (y-axis) and the total number of permutations (x-axis), where each solution is represented with the number of permutations unique by sequence (green points) and by mass (red points). **b** 2D Pareto front of the best, i.e., near optimal solutions (green dots) and all the remaining solutions (blue dots) from the final generation representing mass diversity (x-axis) and sequence diversity (y-axis) relative to the total number of permutations. **c** Sequence logo of library design encircled in subfigure (b). Despite lower mass diversity (81%), the library design maintains high sequence diversity (98%), making it an attractive synthetic possibility. **d**–**f** Refer to the optimization results for the r = 5, m = 7 for $x_i={s,e,r,w,a,G,i}$; $x_3=p$, $x_7=y$ library design

### **Three-objective GA to maximize the complexity through sequence diversity**

Among the permutations with overlapping masses taken from the examples examined in the previous section (Fig. 4 and Additional file 1: Figs. S1–S5), some entries exhibited different amino acid composition (Additional file 1: Fig. S7). In order to preserve these solutions in the library design, we introduced a third fitness function to distinguish the permutations by their composition. Consequently, the optimization implemented for combinatorial peptide library design computed the number of peptides of unique amino acid composition and maximized their sequence diversity. In addition, the introduction of sequence diversity as a fitness function enables the 3-objective GA to find new solutions that may have been neglected in the 2-objective setting because of lower mass diversity. An example is a heptapeptide library from the $x_i={s,e,r,w,a,G,i}$, $x_3={p}$, $x_7={y}$ optimization where two permutations with different sequence, aGpGery and rGpGisy, show monoisotopic masses of 748.3505 and 748.3869, respectively (Additional file 1: Fig. S7). In this particular case, the monoisotopic mass of ‘*ae*’ dipeptide (218.116) overlaps with the mass of the ‘*is*’ dipeptide (218.079). These two peptides would have been excluded in the previous version of the algorithm with $T(\Delta mass)=1$, but now they are included because of the increased sequence diversity within the library.

Using the 3-objective GA, the optimization results are displayed in a three-dimensional Pareto front, from which the 2D projections of both amino acid diversity and mass diversity can be extracted and analysed separately (Additional file 1: Fig. S6). In addition to the output obtained with the 2-objective GA, the output of the 3-objective optimization contains a separate csv file listing all the permutations unique by sequence. In this setting, we performed the optimization for two libraries: r = 5, m = 6 for $x_i={s, e, r, w, a, G}$; $x_6=y$ in Fig. 5a–c and r = 5, m = 7 for $x_i={s,e,r,w,a,G,i}$; $x_3=p$, $x_7=y$ in Fig. 5d–f. We analyzed the output of these examples showing the difference between the sequence and mass diversity of the best solutions, followed by the analysis of the 70% to 100% diversity region of all solutions and finally presenting the sequence logo graphs of two prominent solutions.

The diversity analysis of the libraries (Fig. 5a, d) consists of a parallel representation of sequence and mass diversity distributions on the y-axis for all the best solutions, where each has a different total number of permutations on the x-axis. In these figures, three distinct regions can be observed:

1. for smaller library sizes, composed of 200 or less permutations, the optimization outputs of mass and sequence diversity overlap;
2. for medium sized libraries, containing a 1000 or more permutations, the sequence diversity exhibits a faster growth than the mass diversity;
3. for large libraries with more than 2000 permutations, both diversities saturate and exhibit almost no growth with the increase of library size.

The behavior of the optimization results is summarized in the optimal solutions graph (Additional file 1: Fig. S6d), where two areas of interest are labeled: the overlapping zone (1) where the diversity by sequence and by mass is very similar, and the diversity zone (2) where the diversity by sequence is greater than the diversity by mass. This underlines the overall advantage of the introduction of the third objective in the optimization process that offers a greater possibility to fine-tune the design and fulfill the users’ requirements. As expected, the number of peptides unique by sequence is higher than the number of peptides unique by mass, but both numbers have a theoretical maximum, which is considerably lower than the total number of permutations within the library.

Figure 5b, e present the 2D graphs of the best solutions (green dots) alongside all the solutions (blue dots) from the optimization representing mass diversity on the x-axis and sequence diversity on the y-axis, relative to the total number of permutations. This representation includes an overview of several other design options available that might be of interest to the user and could lead to highly diverse libraries. We focused our interest to the 70% to 100% diversity region and chose only solutions of sequence diversity higher than 95% even if their mass diversity was lower. Two prominent designs are marked and their sequence logo graphs presented in Fig. 5c, f. Both the examples show sequence diversity of 98% while their diversity by mass is 89% and 81%, respectively. Considering that the sequence diversity is a stronger indicator of chemical diversity within the library, it is evident that these two solutions cover a wide range of chemical properties and possibly offer alternative design choices worth of consideration.

In the current system, if the user is interested in making a hydrophobic library, the input should consist of preferentially hydrophobic amino acids. The algorithm-assisted, three-objective optimization offers different possibilities of peptide designs, shown for the high diversity region (70–100%) as well as for the 10–50% diversity region, in Additional file 1: Figs. S8 and S9.

### **Comparison of two- and three-objective settings and the effect of**$\mathbf T (\Delta \mathbf mass)$

**Fig. 6:**
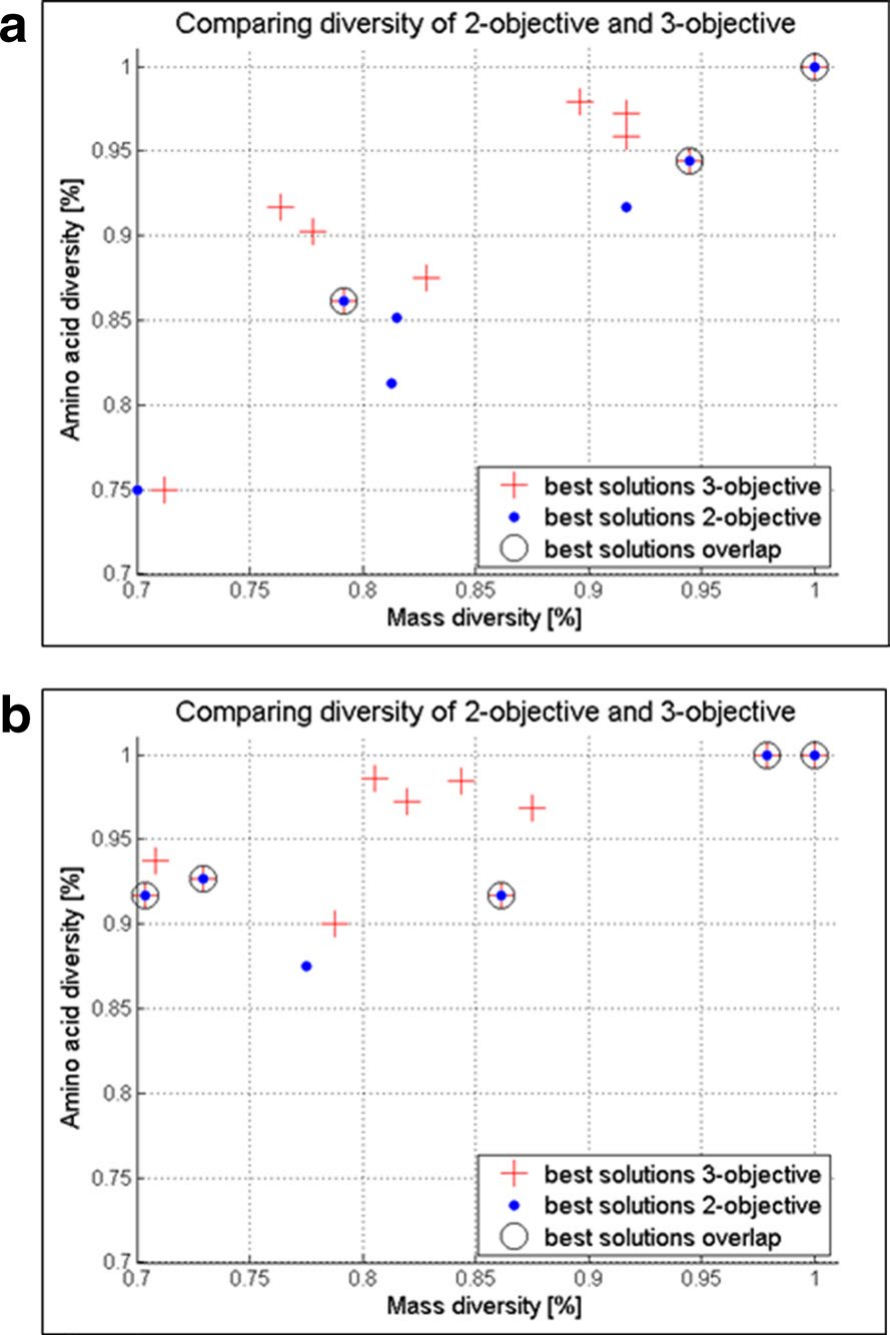
3-objective versus 2-objective comparison. Parallel representation of the best solutions obtained by 2-objective (blue dots) and 3-objective (red crosses) optimization marking the overlapping solutions (circled) for the same input data in terms of mass diversity (x-axis) and sequence diversity (y-axis) relative to the total number of permutations for **a** r = 5, m = 6 and $x_i={s, e, r, w, a, G}$; $x_6=y$ library design and for **b** r = 5, m = 7 and $x_i={s,e,r,w,a,G,i}$; $x_3=p$, $x_7=y$ library design

**Fig. 7:**
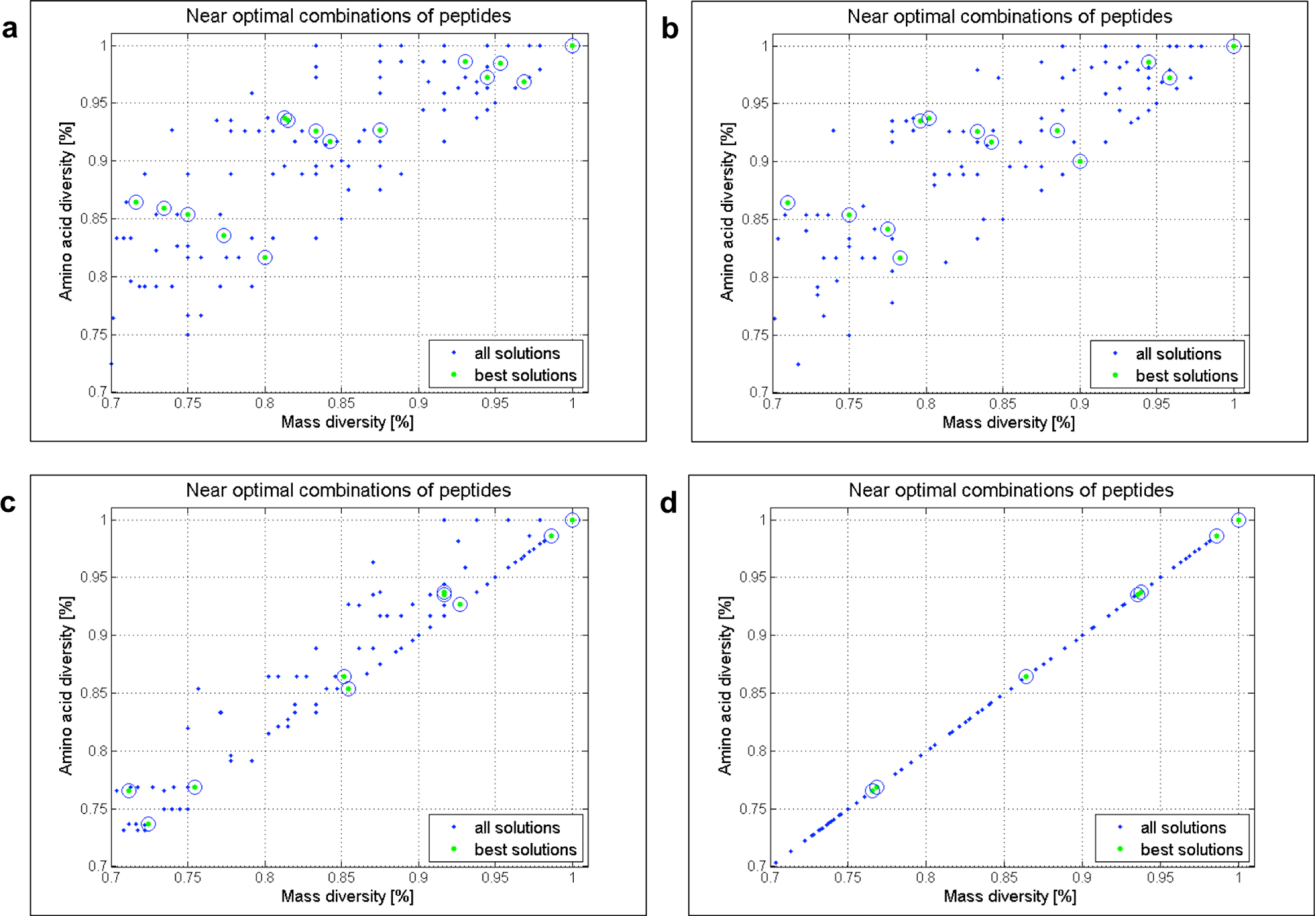
The effect of varying $T(\Delta mass)$ on the distribution of best solutions. The analysis of mass diversity (x-axis) and sequence diversity (y-axis) of the best, i.e., near optimal solutions (green dots) and all the remaining solutions (blue dots) is given for **a** $T(\Delta mass) = 0.5$, **b** $T(\Delta mass) = 0.1$, **c** $T(\Delta mass) = 0.01$ and **d** $T(\Delta mass) = 0.001$

Although the computational cost for the three-objective GA optimization is higher, the benefit of increasing the sequence diversity is visible when comparing the output results, i.e., the best solutions of the 3- and 2-GA optimizations. Figure 6 presents this comparison in the 70% to 100% diversity region for two different inputs: (a) r = 5, m = 6 and $x_i={s, e, r, w, a, G}$; $x_6=y$ and (b) r = 5, m = 7 and $x_i={s,e,r,w,a,G,i}$; $x_3=p$, $x_7=y$. It can be noticed that the best solutions from the 3-objective optimization (red crosses) are more numerous than the 2-objective ones (blue dots), indicating the coverage of a larger search space and greater design choices. However, the number of solutions with higher diversity is closely related to the chosen value of the tolerance of mass discrimination $T(\Delta mass)$. As previously mentioned, we set this parameter to $T(\Delta mass) = 1$ to discriminate the permutations by mass. Therefore, should a user require lower or higher tolerance values, the ratio between the number of permutations unique by sequence and unique by mass would change accordingly.

As expected, some of the proposed designs overlap in both optimizations as shown in Fig. 6a, b. This suggests that the choice of the optimization method will depend solely on the users’ requirements. When the mass diversity design is sufficient, the 2-objective optimization will be the method of choice. Should a user require additional design suggestions based on sequence diversity, it can opt for the more costly but more informative 3-objective method.

**Fig. 8:**
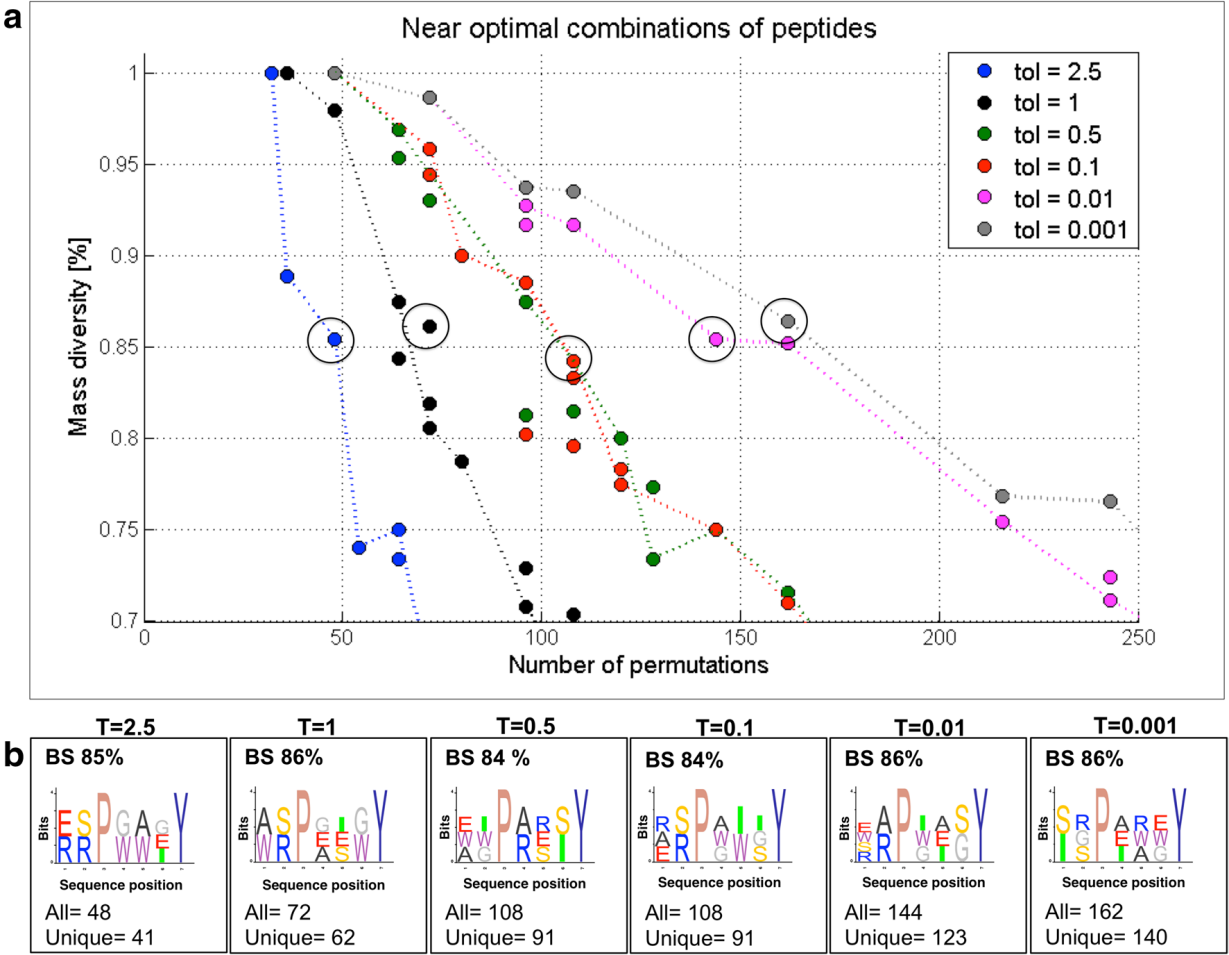
The effect of varying $T(\Delta mass)$ on the number of permutations. **a** Parallel representation of pareto fronts in the 70–100% diversity range, for $T(\Delta mass) \in \left\{ 2.5, 1, 0.5, 0.1, 0.01, 0.001 \right\}$ obtained by the 3-objective optimizations for (r = 5, m = 7 and $x_i={s,e,r,w,a,G,i}$; $x_3=p$, $x_7=y$) library design, where each optimization run is presented in different color. **b** Sequence logos of library designs encircled in subfigure **a**

Next, we explored the effect of varying the tolerance input to values of $T<1$ and $T>1$ for the optimization described in Fig. 4 using both, the two- (Additional file 1: Figs. S10–S14) and the three-objective (Figs. 7, 8 and Additional file 1: S14) settings. When changing $T(\Delta mass)$ to 2, 5, 0.5, 0.1, 0.01 and 0.001, the distribution of best solutions (Fig. 7) and the number of permutations for related mass diversities are affected (Fig. 8). The impact of varying the $T(\Delta mass)$ from 0.001 to 2.5 on the number of best solutions is visible in the optimization results where a smaller tolerance window yields a lower number of best solutions. An example is the high diversity region (sequence diversity above 90%), where the number of best solutions is 10 for T = 0.5, 9 for T = 0.1, 5 for T = 0.01 and only 4 for T = 0.001 (Fig. 7). In addition, when lowering $T(\Delta mass)$ to values close to zero such as 0.001 and 0.01, the algorithm behaves as sequence diversity was the measure of library diversity, i.e. it works according to the 3rd objective. This results in mass and sequence diversity being linearly correlated as seen in Fig. 7d.

In agreement with the spread of pareto fronts shown in Fig. 8, the number of permutations increases for decreasing values of $T(\Delta mass)$ and hence, the pareto front shifts to the right accordingly. By comparing best solutions having 85($\pm 1$)% mass diversity (Fig. 8), it can be noticed that the number of permutations increases from 48 (T = 2.5) to 72 (T = 1), 108 (T = 0.5 and T = 0.1), 144 (T = 0.01) and 162 (T = 0.001). For $T(\Delta mass)$ values below 1, i.e., 0.5, 0.1, 0.01 and 0.001, no differences are observed in terms of number of library components for BS1 (100% diversity). On the other hand, when increasing the $T(\Delta mass)$ value to 1 and 2.5, BS 1 (100% diversity) shows a decreasing number of library components from 36 (BS1, $T=1$) to 32 (BS1, $T=2.5$).

Furthermore, in the 2-objective setting, by lowering the tolerance window, the number of permutations with unique mass increases, to take into account those sequences that have different amino acid composition but mass difference lower than $T(\Delta mass)$. As expected, there is little difference in optimization results between $T(\Delta mass)$ values of 0.5 and 0.1. In the high diversity region BS1, BS2, BS3 and BS5 match while BS 4 shows one more peptide unique by mass for $T=0.1$. When looking at $T=0.01$, the differences are more pronounced in terms of mass diversity and number of library components seen for BS 2, BS 3, BS 4 and BS 5 when compared to $T(\Delta mass)$ values of 0.5 and 0.1 (Additional file 1: Figs. S10–S13).

## Conclusions

When dealing with collections of structurally similar compounds, such as peptides that have the same amino acid composition but differential positioning of residues in the sequence (permutations), similar or identical mass, and similar physico-chemical properties, it is challenging to discriminate single permutations with high accuracy. Therefore, diversity rather than library size is the key element when designing random peptide libraries for the discovery of novel biologically active peptides.

In this paper, we have presented an algorithm-supported methodology for the design of random peptide libraries. Basing the methodology on the multi-objective genetic algorithms we achieved libraries with maximal number of peptides that seek to maximal mass and/or sequence diversity. In the two-objective setting, where the goal was mass diversity, the tolerance parameter allows the library designer to define how different two peptides should be in terms of their mass. Moreover, sequence diversity was achieved in the three-objective setting that offered additional library solutions whit similar mass but differential composition of amino acids. This system could be extended to several other fitness functions and their combinations. A possible future multi-objective GA could take into account the charge or the hydrophobicity/philicity of the library components.

Intelligent systems that operate under controlled conditions allow us to explore huge search spaces and offer a large number of possible solutions. It is up to the user to choose the solution of interest. Throughout a series of examples we highlighted the advantage of having numerous design suggestions before attempting any synthesis of complex mixtures. In this way, we could rationally design a library or different pools of smaller libraries—having desired properties and amino acid compositions—based on informed and careful user choices. Thus, this paper demonstrated the necessity of interlinking the advanced computing capabilities of genetic algorithms and the design of peptide libraries. The size of the search space makes heuristic algorithms the method of choice. In fact, the range of possible solutions is difficult to access to an expert user even for smaller scale inputs.

## Materials and methods

The definition of input parameters is presented in Algorithm 1, where *r* is the number of positions, *counter* is the number of amino acids at each position and *full list* is the full amino acid list:

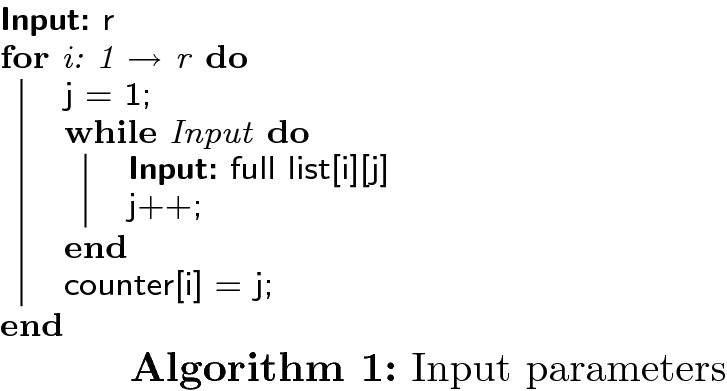



The core NSGA-II algorithm is used from the Matlab built-in script *gamultiobj*. Its default parameters are modified as follows:

- *‘PopulationSize’* is set to 500,
- *‘ParetoFraction’* is set to 0.2,
- *‘PopulationType’* is set to ‘bitstring’.

**Fig. 9:**
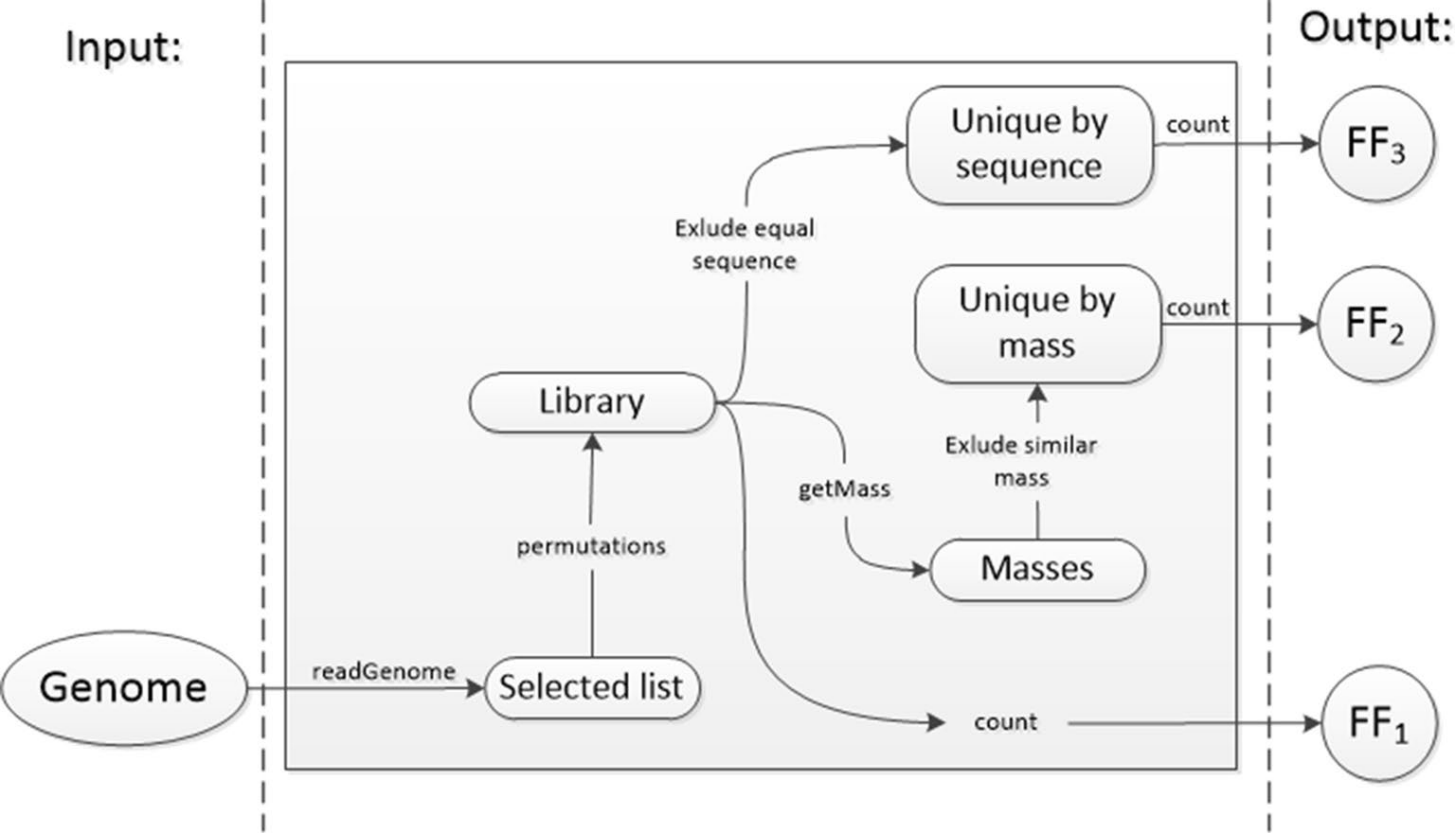
**Schematic representation of the computation of 3 objectives within the NSGA-II**

The binary encoded genome for NSGA-II is created as a bit-string of length:

1 $$\begin{aligned} length = \sum _{i=1}^{r} counter[i] \end{aligned}$$

The methodology used to compute the three fitness functions ($FF_1$, $FF_2$ and $FF_3$) for each *Genome* is presented graphically in Fig. 9.

Algorithm 2 (*readGenome*) is used to transform the genome of a solution obtained by NSGA-II into a algorithm selected list of amino acids (*selected_list*):

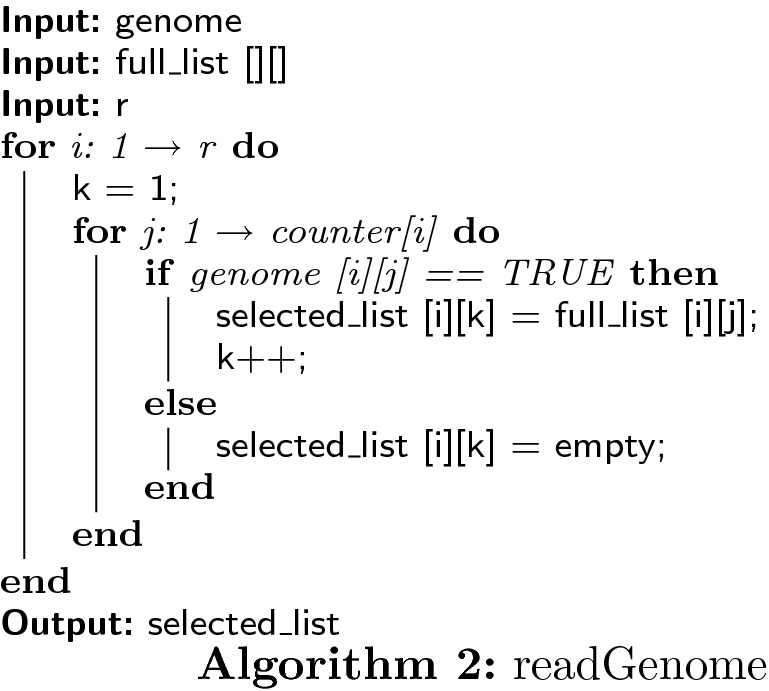



The analysis of the library solution (*selected_list*) is followed by the computation of the permutations which constitute the *Library*. Algorithm *permutations* uses Matlab built-in function *ndgrid* to make the computation.

Algorithm (*getMass*) is used to compute the mass of every permutation within the *Library* using the following equation:

2 $$\begin{aligned} M = \left[ \sum _{i=1}^{r} m(a_i) \right] - (r - 1) \cdot m(H_2 O) \end{aligned}$$

where *M* represents the mass of a peptide, $m(a_i)$ represents the mass of amino acid *a* at position *i* within the peptide, and $m(H_2 O)$ is the mass of a molecule of water. Slightly modifying the Eq. 2, the algorithm computes the average mass, the monoisotopic mass, the singly charged $[M+H]^+$ or the doubly charged $[M+2H]^{2+}$ mass of peptides.

The analysis of the *Library* is followed by the exclusion of peptides of similar mass implemented in Algorithm 3:

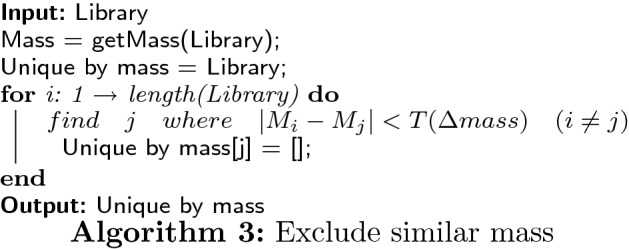



where $M_i$ and $M_j$ are masses of permutations at positions *i* and *j* within the Library, while $T(\Delta mass)$ is the tolerance of mass discrimination. If the absolute value of mass difference between permutations at positions *i* and *j* is less than $T(\Delta mass)$, then the permutation at position *j* is removed from the *Library* list. Algorithm *Exclude similar mass* repeats this process until all the permutations are compared and only the ones with unique mass remain in the list (*Unique by mass*).

The analysis of the *Library* is also followed by the exclusion of peptides of equal sequence implemented in Algorithm 4:

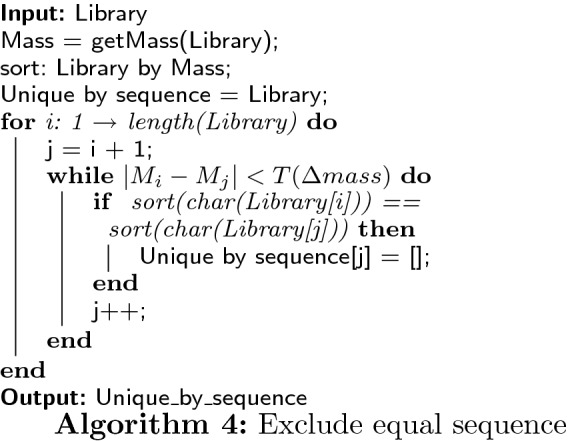



The algorithm *Exclude equal sequence* uses the algorithm *getMass* to speed up the search because peptides of similar mass are candidates for peptides of equal sequence. The candidate permutations within the *Library* need to be transformed into a list of characters and sorted alphabetically. If two peptides have equal list of sorted characters, one of them is excluded from the *Library* list. Algorithm *Exclude equal sequence* repeats this process until all the permutations are compared and only the ones with unique sequence remain in the list (*Unique by sequence*).

## Additional files

**Additional file 1.** Supporting information for Algorithm-supported, mass and sequence diversity-oriented random peptide library design

**Additional file 2.** List of amino acid permutations for BS 2, from the optimization in Fig. 4.

## Abbreviations

**GA**: genetic algorithm
**NSGA-II**: non-dominated solutions genetic algorithm II
**csv**: comma-separated values
**BS**: best solution
**MW**: molecular weight
**OBOC**: one-bead-one-compound
**DOS**: diversity-oriented systems
**HPLC**: high performance liquid chromatography
**UPLC-MS**: ultra-high performance liquid chromatography-mass spectrometry
**BBB**: blood-brain-barrier
**MHC**: major histocompatibility complex
