# Supplementary material for: Algorithm-supported, mass and sequence diversity-oriented random peptide library design
Source: J Cheminform. 2019 Mar 28;11:25. doi: 10.1186/s13321-019-0347-6 (PMC6437963; doi:10.1186/s13321-019-0347-6)
Supplement: Supplementary file 1 — Additional file 1. Supporting information for Algorithm-supported, mass and sequence diversity-oriented random peptide library design [file 13321_2019_347_MOESM1_ESM.pdf]

Supporting information

Algorithm-supported, Mass and Sequence Diversity-oriented Random Peptide Library Design

Daniela Kalafatovic[1]*, Goran Mauša[2], Toni Todorovski[1], Ernest Giralt[1,2]

[1] Institute for Research in Biomedicine (IRB Barcelona), The Barcelona Institute of Science and Technology (BIST), Baldiri Reixac, 10, 08028 Barcelona, Spain

[2] Faculty of Engineering, University of Rijeka, Vukovarska 58, 51000, Rijeka, Croatia

[3] Department of Inorganic and Organic Chemistry, University of Barcelona, Marti i Franques, 1-5, 08028 Barcelona, Spain

* Correspondence should be sent to daniela.kalafatovic@gmail.com

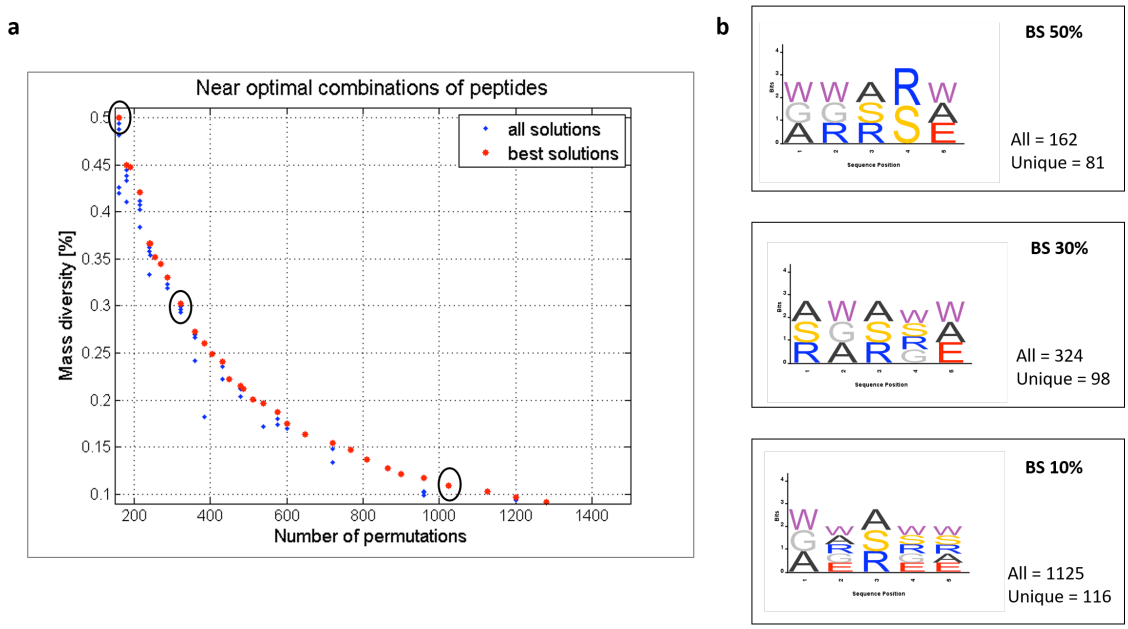

Fig. S1 (a) Pareto front zoom, range: <10% - 50%> of the optimization results for the OBOC peptide library having 5 positions where variability was introduced (r=5), with *m=6* and x_i=*{s,e,r,w,a,G}*. We chose three best solutions: BS 50%, BS 30% and BS 10%. **(b)** Sequence logo representations of BS 50%, BS 30% and BS 10%.

a

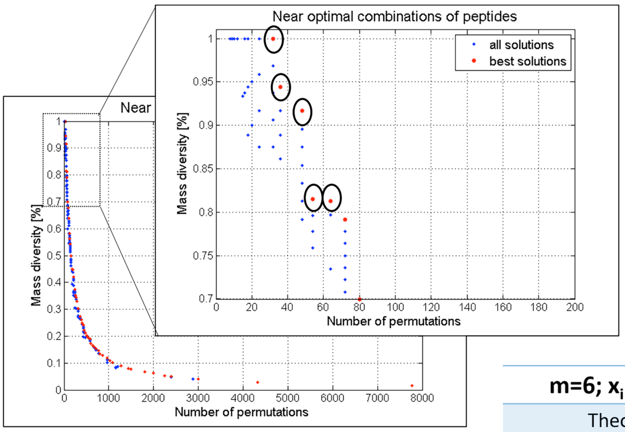

b

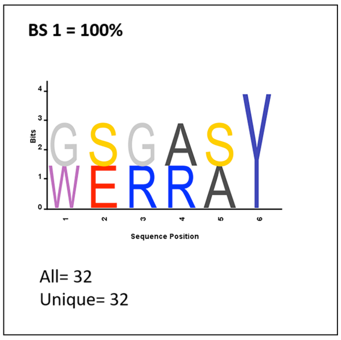

$m=6$; x_i = {s, e, r, w, a, G}; x_6=y	r = 5
Theoretical permutations n	7776
Search space = 2^(5*6)	1.07 * 10^9

c

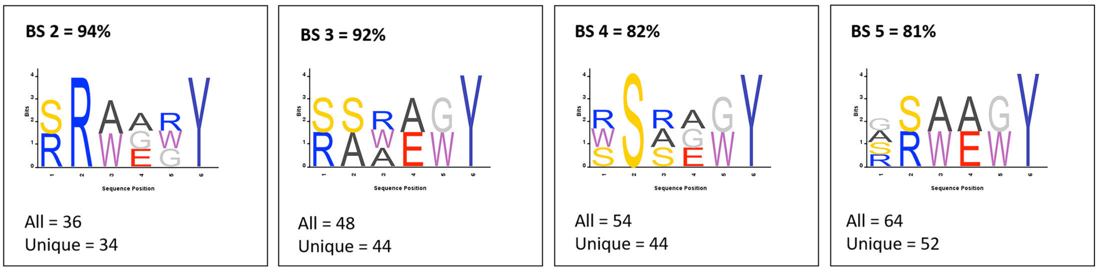

Fig. S2 (a) Pareto front (output) of the optimization results for the OBOC peptide library having 5 positions where variability was introduced (r=5), with *m=6* and x_i=*{s,e,r,w,a,G,}* and one fixed position, being x_6=y. In the zoom of the pareto front, in the range: <70% - 100%>, we chose five best solutions: BS 1 (100%), BS 2 (94%), BS 3 (92%), BS 4 (82%) and BS 5 (81%). **(b)** Sequence logo representation of the BS 1, showing the design we would propose for further examination. **(c)** Sequence logos of BS 2, BS 3, BS 4 and BS 5 suggesting various synthetic possibilities and pointing out possible synthetic challenges. Several other design suggestions are available, but we show only these five for simplicity.

a

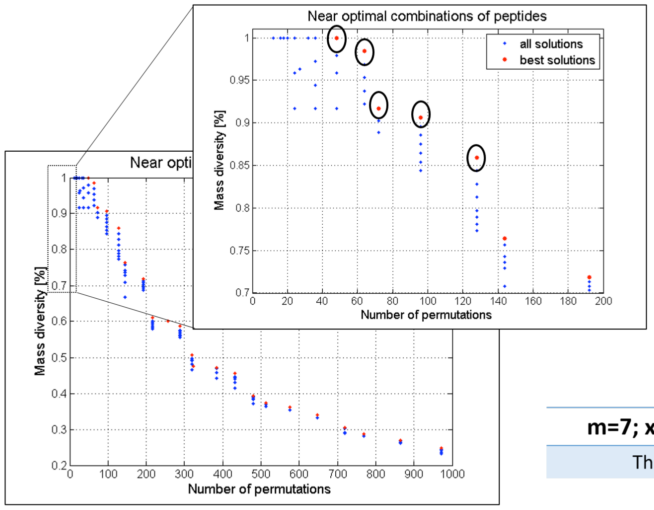

b

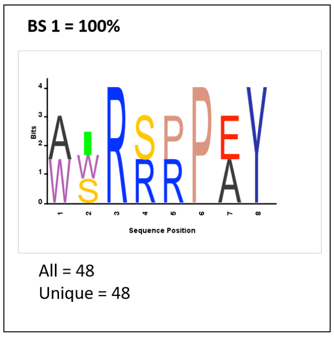

m=7; x_i = {w, r, e, p, s, i, a}; x_7=y	r = 7
Theoretical permutations n	823,543
Search space = 2^(7*7)	5.63 * 10^{14}

c

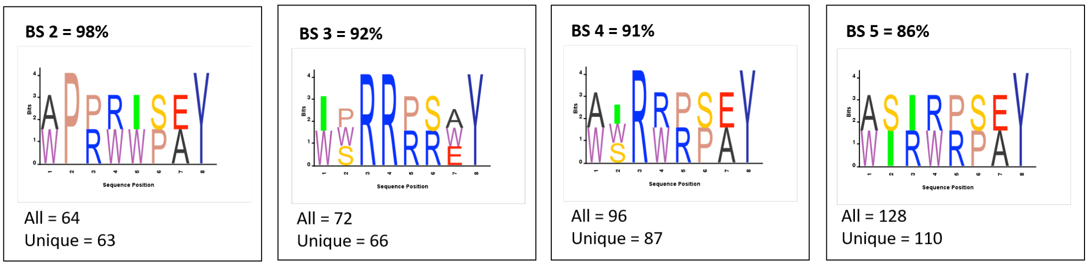

Fig. S3 (a) Pareto front (output) of the optimization results for the OBOC peptide library having 7 positions where variability was introduced (r=5), with m=7 and x_i={w,r,e,p,s,i,a} and one fixed position, being x_8=y. In the zoom of the pareto front, in the range: <70% - 100%>, we chose five best solutions: BS 1 (100%), BS 2 (98%), BS 3 (92%), BS 4 (91%) and BS 5 (86%). **(b)** Sequence logo representation of the BS 1, with the total number of permutations if all the 7 amino acids were used in all the 7 positions alongside the search space accessed by the algorithm. **(c)** Sequence logos of BS 2, BS 3, BS 4 and BS 5 suggesting various synthetic possibilities and pointing out possible synthetic challenges. As for previous examples, several other design suggestions are available.

a

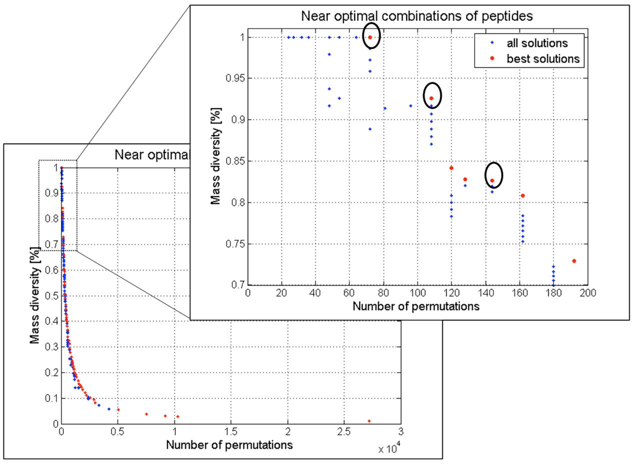

b

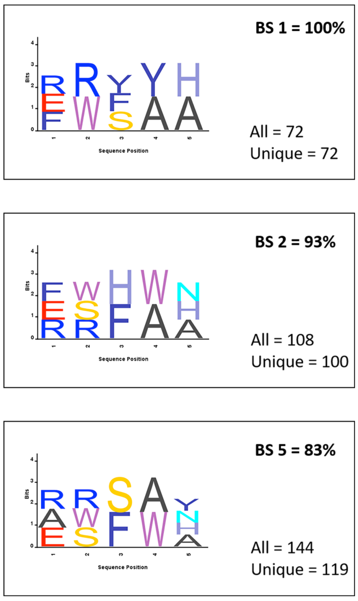

m=10; x_i = {h, f, r, a, n, e, s, y, w, i}	r = 5
Theoretical permutations n	100,000
Search space = 2^(5*10)	1.12 * 10^{15}

Fig. S4 (a) Pareto front (output) of the optimization results for the OBOC peptide library having 5 positions where variability was introduced (r=5), with *m=10* and x_i*={h,f,r,a,n,e,s,y,w,i}*. In the zoom of the pareto front in the range: <70% - 100%>, we chose three best solutions: BS 1 (100%), BS 2 (93%) and BS 5 (83%) suggesting various synthetic possibilities and pointing out possible synthetic challenges. In addition, the total number of permutations if all the 10 amino acids were used in all the 5 positions alongside the search space accessed by the algorithm are shown.

a

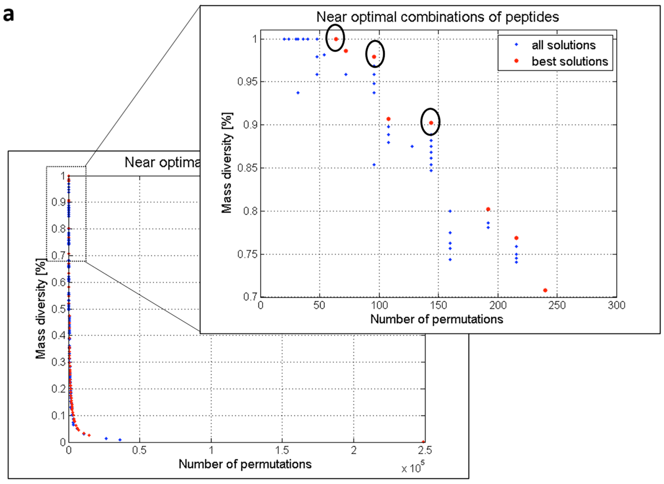

b

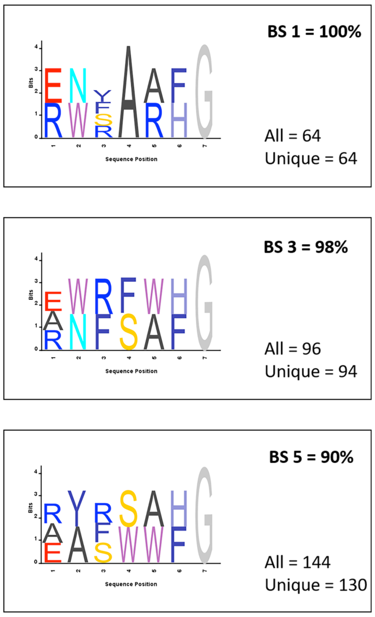

x=10; x_i = {h, f, r, a, n, e, s, y, w, i}; x_7=G	r = 6
Theoretical permutations n	1,000,000
Search space = 2^(6*10)	1.15 * 10^{18}

Fig. S5 (a) Pareto front (output) of the optimization results for the OBOC peptide library having 6 positions where variability was introduced (r=6), with *m=10* and x_i*={h,f,r,a,n,e,s,y,w,i}* and one fixed position x_7*=G*. In the zoom of the pareto front in the range: <70% - 100%>, we chose three best solutions: BS 1 (100%), BS 3 (98%) and BS 5 (90%) suggesting various synthetic possibilities and pointing out possible synthetic challenges. In addition, the total number of permutations if all the 10 amino acids were used in 6 positions alongside the search space accessed by the algorithm are shown.

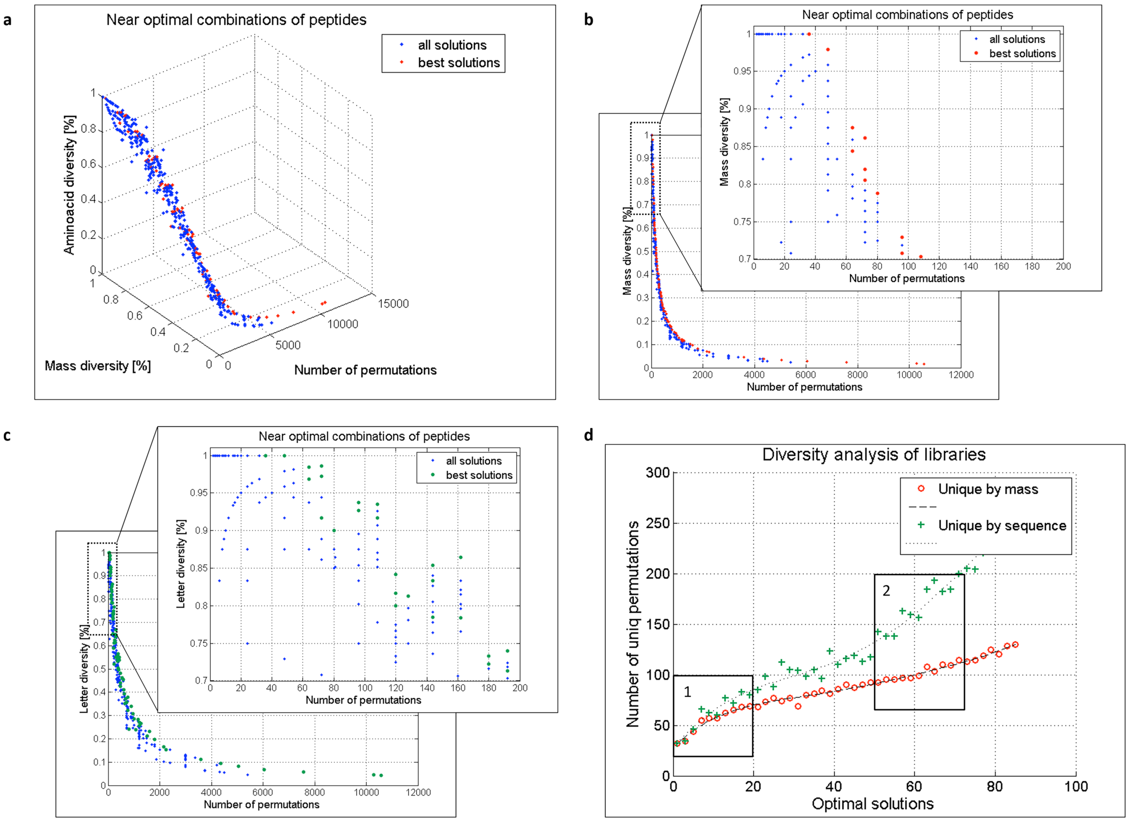

Fig. S6 (a) 3D Pareto front of the best, i.e. near optimal solutions (red dots) and all the remaining solutions (blue dots) from the final generation, calculated with the 3-objective optimization for the following input: the number of positions, with 5 variable positions (r=5) with m=7 and x_i=*{s,e,r,w,a,G,i}*), 2 fixed positions (x_3=*{p}*, x_7=*{y}*) and tolerance (Δmass=1) to discriminate between permutations. **(b)** 2D Pareto front representing mass diversity (y-axis) relative to the total number of permutations (x-axis) with zoomed presentation in the range 70% ≤ mass diversity ≤100%. **(c)** 2D Pareto front representing sequence diversity (y-axis) relative to the total number of permutations (x-axis) with zoomed presentation in the range 70% ≤ sequence diversity ≤100%. **(d)** Diversity analysis of all best solutions for library design (x-axis) in terms of the number of unique permutations (y-axis), where each solution is represented with the number of permutations unique by sequence (green points) and by mass (red points). Two areas of interest are labeled with: (1) Overlapping zone where the diversity by sequence and by mass is very similar, (2) Diversity zone where the diversity by sequence is greater than the diversity by mass.

Unique by sequence

Entry	Permutations	Monoiso_mass	[M+H]
1	aGpGssy	637.2708	638.2786
2	aGpGisy	663.3228	664.3306
3	aGpGesy	679.2813	680.2892
4	aGpGsry	706.34	707.3478
5	aGpessy	709.2919	710.2997
6	rGpGssy	722.3348	723.3427
7	aGpGiry	732.392	733.3998
8	aGpeisy	735.3439	736.3517
**9**	**aGpGery**	**748.3505**	**749.3583**
**10**	**rGpGisy**	**748.3869**	**749.3947**
11	aGpeesy	751.3024	752.3103
12	wGpGssy	752.313	753.3208
13	rGpGesy	764.3454	765.3532
14	awpGssy	766.3286	767.3365

Unique by mass

Entry	Permutations	Monoiso_mass	[M+H]
1	aGpGssy	637.2708	638.2786
2	aGpGisy	663.3228	664.3306
3	aGpGesy	679.2813	680.2892
4	aGpGsry	706.34	707.3478
5	aGpessy	709.2919	710.2997
6	rGpGssy	722.3348	723.3427
7	aGpGiry	732.392	733.3998
8	aGpeisy	735.3439	736.3517
9	aGpGery	748.3505	749.3583
10	aGpeesy	751.3024	752.3103
11	wGpGssy	752.313	753.3208
12	rGpGesy	764.3454	765.3532
13	awpGssy	766.3286	767.3365
14	aGpesry	778.361	779.3689

Fig. S7 Screenshot of a part of the list of permutations unique by sequence (left) and unique by mass (right) highlighting the mass overlapping of two permutations that differ by amino acid composition but were excluded in the unique by mass 2-objective optimization. In this particular case the monoisotopic mass of 'ae' dipeptide (218.116) overlaps with the one of the 'is' dipeptide (218.079).

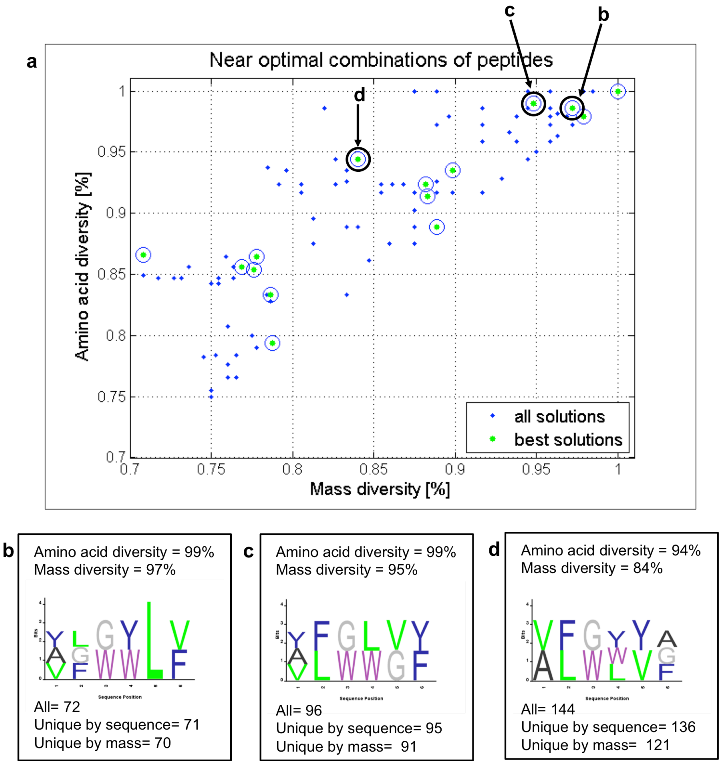

Fig. S8 (a) 2D Pareto front of the best, *i.e.* near optimal solutions (green dots) and all the remaining solutions (blue dots) from the final generation representing mass diversity (x-axis) and sequence diversity (y-axis) relative to the total number of permutations. **(b)**, **(c)** and **(d)** Sequence logos of library designs encircled in subfigure (a) for *r=6*, *m=7* for *xi={f,w,y,l,a,v,G}* and *T(Δmass)=0.1* input.

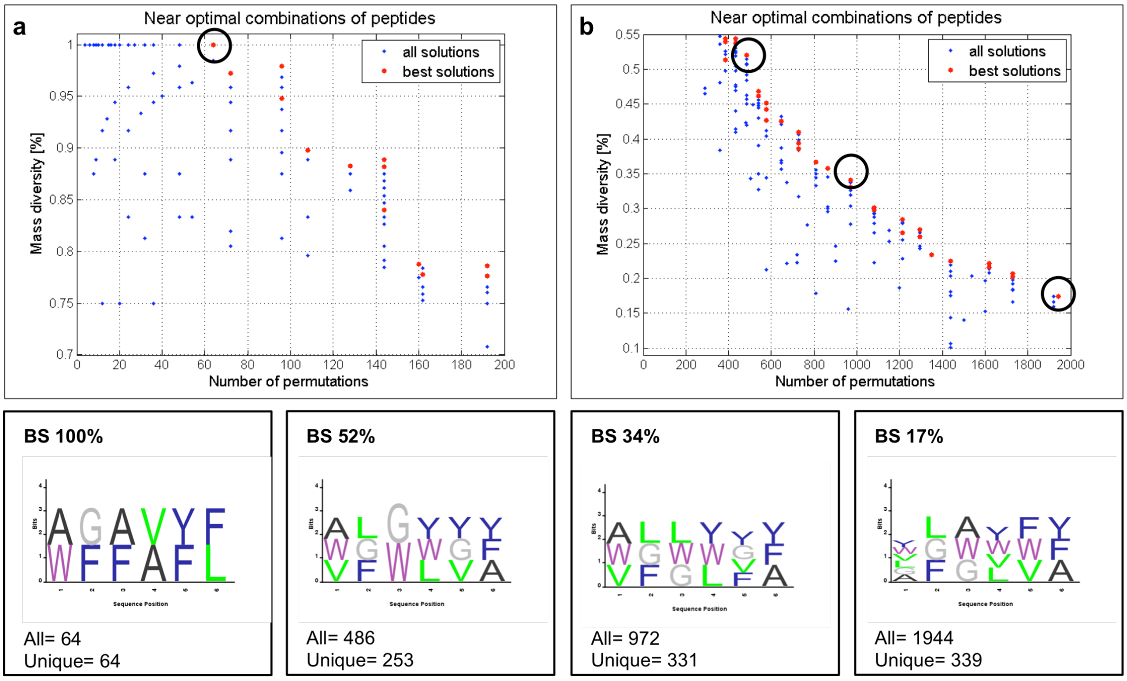

Fig. S9 Pareto front zoom, range: **(a)** <70% - 100%> and **(b)** <10% - 50%> of the optimization results for the OBOC peptide library having 6 positions where variability was introduced *(r=6)*, with *m=7* for x_i=*{f,w,y,l,a,v,G}* and *T(Δmass)=0.1*. Sequence logo representations of four best solutions are presented below: BS 100%, BS 52%, BS 34% and BS 17%.

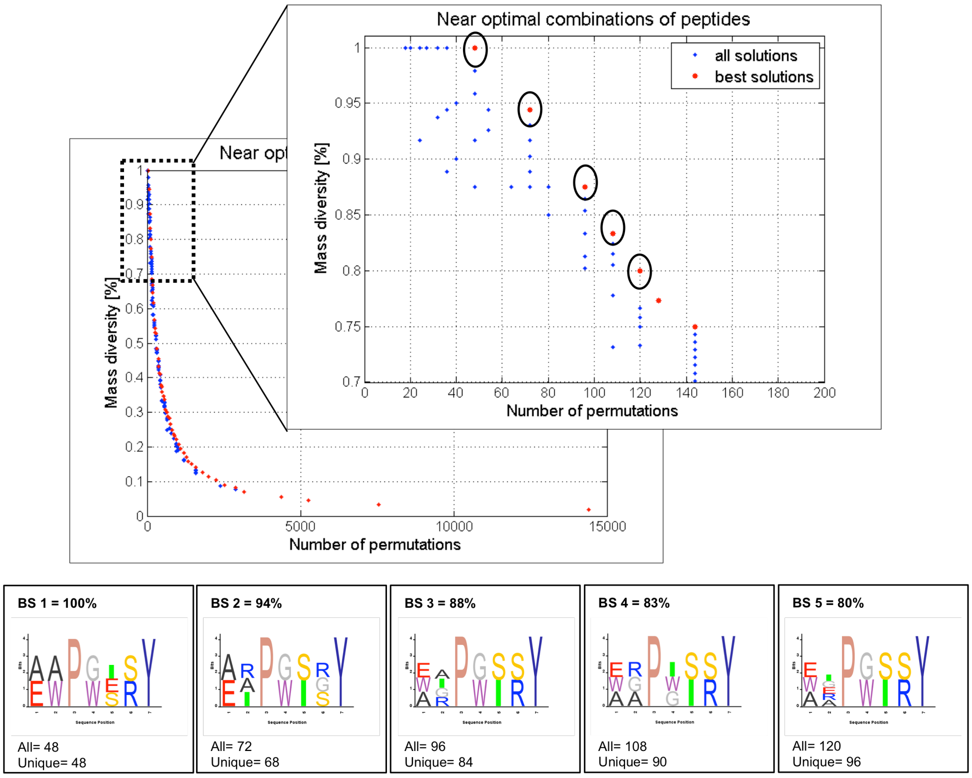

Fig. S10 Tollerance=0.5. Pareto front of the optimization results for the OBOC peptide library from figure 2 (r=5, *m*=7 for x_i={s,e,r,w,a,G,i} and x_3=p, x_7=y) with *T(Δmass) set to 0.5*. In the zoom of the pareto front, in the range: <70% - 100%>, we chose five best solutions: BS 1 (100%), BS 2 (94%), BS 3 (88%), BS 4 (83%) and BS 5 (80%) and presented their sequence logos. As for previous examples, several other design suggestions are available.

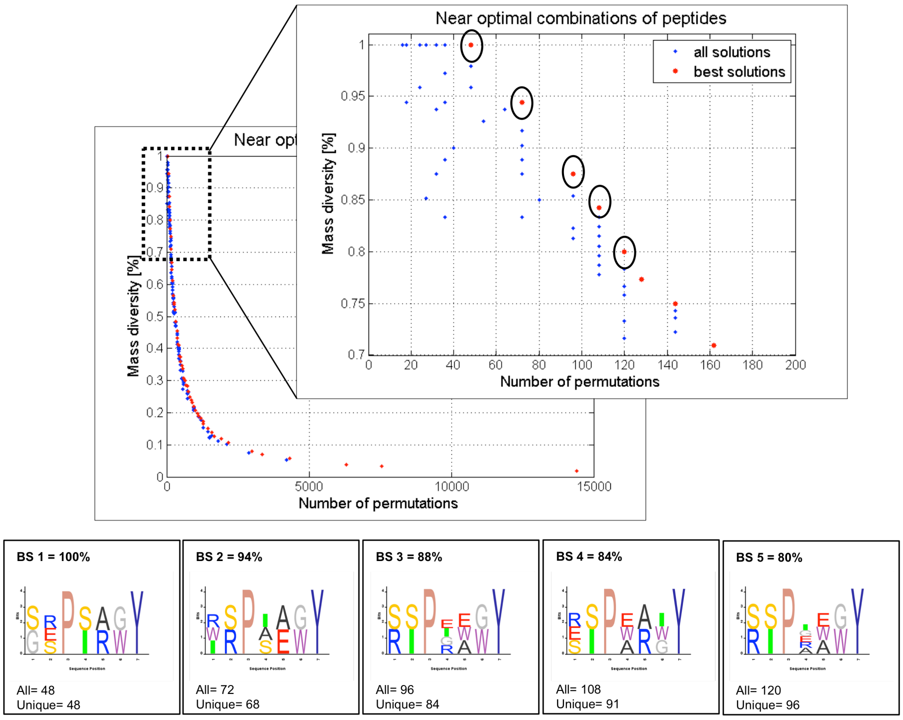

Fig. S11 Tollerance=0.1. Pareto front of the optimization results for the OBOC peptide library from figure 2 (r=5, *m*=7 for x_i={s,e,r,w,a,G,i} and x_3=p, x_7=y) with *T(Δmass) set to 0.1*. In the zoom of the pareto front, in the range: <70% - 100%>, we chose five best solutions: BS 1 (100%), BS 2 (94%), BS 3 (88%), BS 4 (84%) and BS 5 (80%) and presented their sequence logos. As for previous examples, several other design suggestions are available.

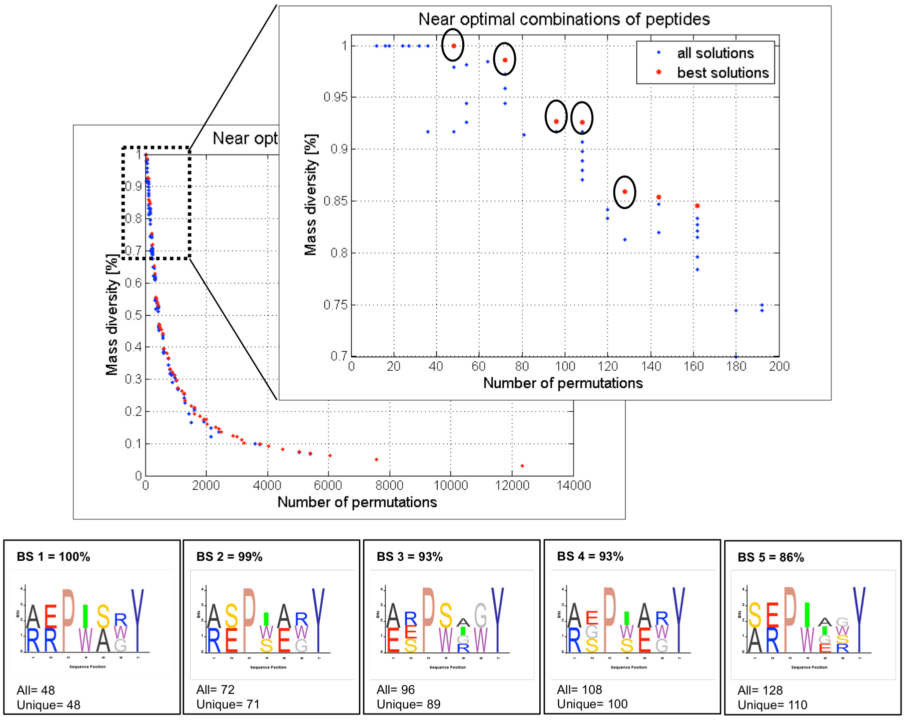

Fig. S12 Tollerance=0.01. Pareto front of the optimization results for the OBOC peptide library from figure 2 (r=5, *m*=7 for x_i={s,e,r,w,a,G,i} and x_3=p, x_7=y) with *T(Δmass) set to 0.01*. In the zoom of the pareto front, in the range: <70% - 100%>, we chose five best solutions: BS 1 (100%), BS 2 (99%), BS 3 (93%), BS 4 (93%) and BS 5 (86%) and presented their sequence logos. As for previous examples, several other design suggestions are available.

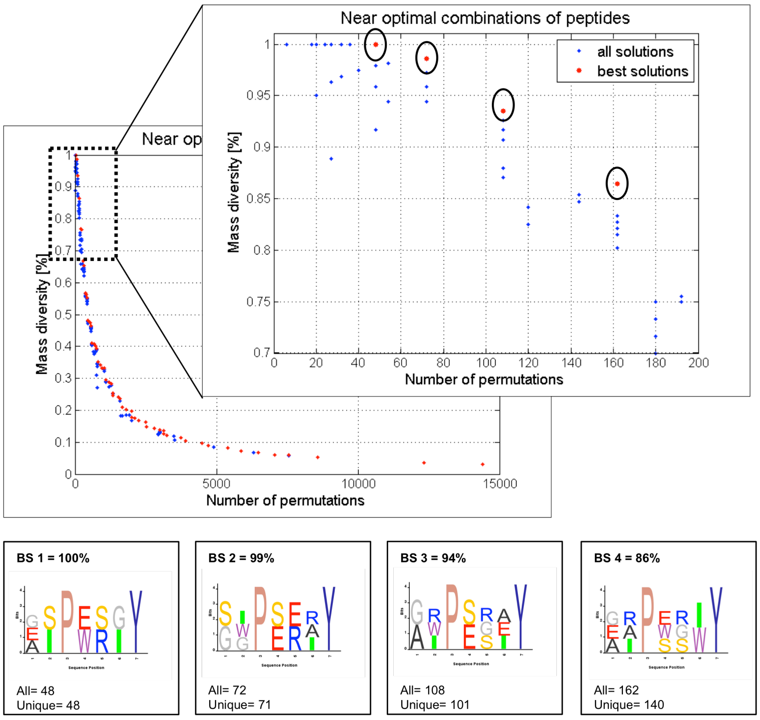

Fig. S13 Tollerance=0.001. Pareto front of the optimization results for the OBOC peptide library from figure 2 (r=5, *m*=7 for x_i={s,e,r,w,a,G,i} and x_3=p, x_7=y) with *T(Δmass) set to 0.001*. In the zoom of the pareto front, in the range: <70% - 100%>, we chose four best solutions: BS 1 (100%), BS 2 (99%), BS 3 (94%), and BS 4 (86%) and presented their sequence logos. As for previous examples, several other design suggestions are available.

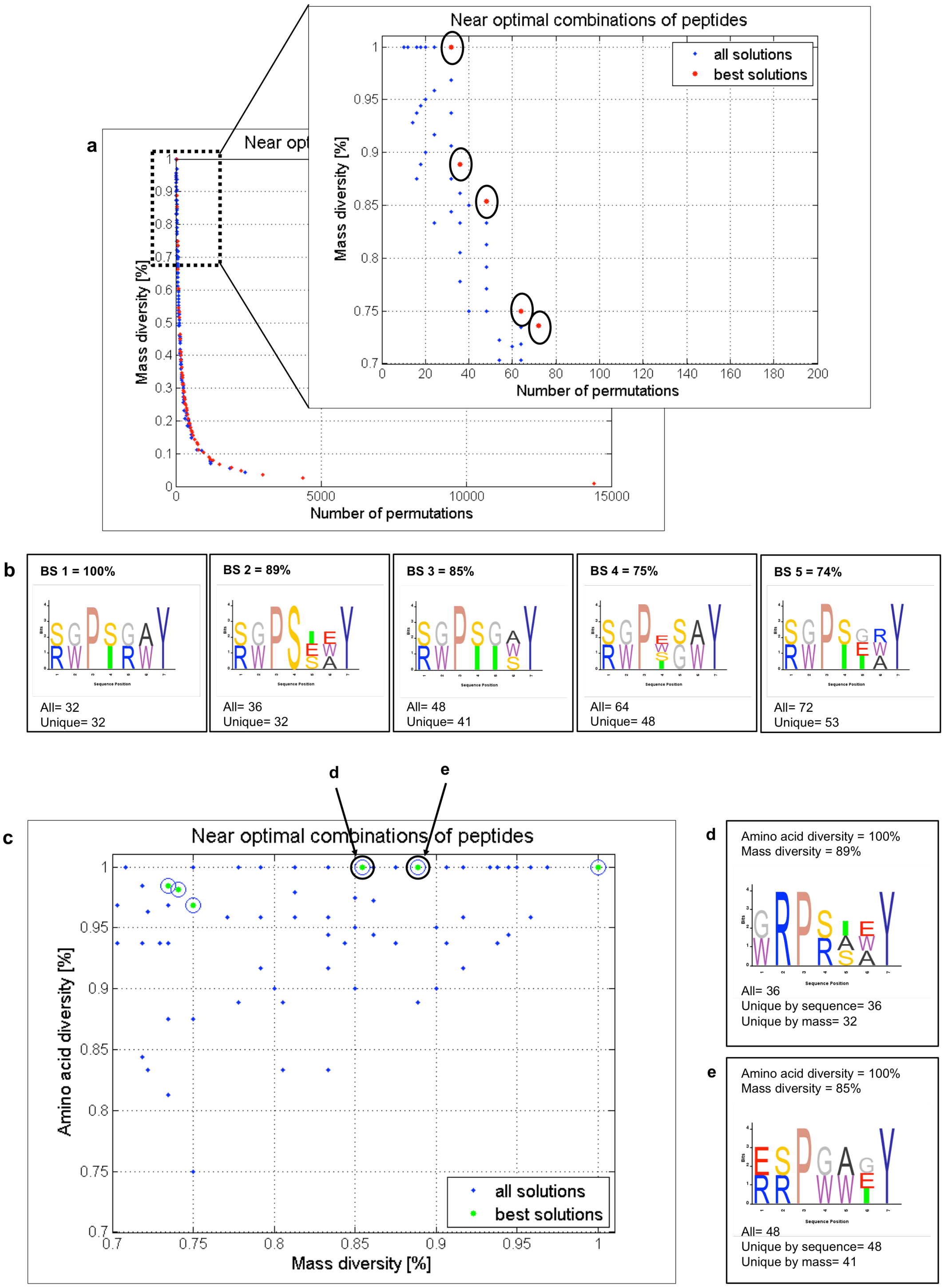

Fig. S14 Tollerance=2.5. (a) Pareto front of the optimization results (2-objective) for the OBOC peptide library from figure 2 (r=5, *m*=7 for x_i={s,e,r,w,a,G,i} and x_3=p, x_7=y) with *T(Δmass) set to 2.5*. In the zoom of the pareto front, in the range: <70% - 100%>, we chose five best solutions: BS 1 (100%), BS 2 (94%), BS 3 (88%), BS 4 (84%) and BS 5 (80%) and presented their sequence logos **(b)**. **(c)** 2D Pareto front of the best, *i.e.* near optimal solutions (green dots) and all the remaining solutions (blue dots) from the final generation representing mass diversity (x-axis) and sequence diversity (y-axis) relative to the total number of permutations (3-objective setting). **(d)** and **(e)** sequence logos of library designs encircled in subfigure (c) for the same input.
