# Supplementary material for: Algorithm-supported, mass and sequence diversity-oriented random peptide library design
Source: J Cheminform. 2019 Mar 28;11:25. doi: 10.1186/s13321-019-0347-6 (PMC6437963; doi:10.1186/s13321-019-0347-6)
Supplement: Supplementary file 2 — Additional file 2. List of amino acid permutations for BS 2, from the optimization in Fig. 4. [file 13321_2019_347_MOESM2_ESM.pdf]

Table S1. List of all the amino acid permutations for BS 2, from the optimization in fig 4.

Entry	Permutations	Average mass	Monoisotopic mass	Expected [M+H]	Expected [M+2H]/2
1	aspasGy	651.6686	651.2864	652.2943	326.6510
2	aspaiGy	677.7492	677.3385	678.3463	339.6771
3	aspaeGy	693.7055	693.2970	694.3048	347.6563
4	aspesGy	709.7050	709.2919	710.2997	355.6538
5	arpasGy	720.7773	720.3556	721.3634	361.1856
6	aspeiGy	735.7856	735.3439	736.3517	368.6798
7	arpaiGy	746.8579	746.4077	747.4155	374.2117
8	aspeeGy	751.7419	751.3024	752.3102	376.6590
9	arpaeGy	762.8142	762.3662	763.3740	382.1909
10	wspasGy	766.8013	766.3286	767.3365	384.1721
11	arpesGy	778.8137	778.3610	779.3689	390.1884
12	aspaswy	780.8279	780.3443	781.3521	391.1800
13	wspaiGy	792.8819	792.3807	793.3885	397.1982
14	arpeiGy	804.8943	804.4131	805.4209	403.2144
15	aspaiwy	806.9085	806.3963	807.4042	404.2060
16	wspaeGy	808.8382	808.3392	809.3470	405.1774
17	arpeeGy	820.8506	820.3716	821.3794	411.1936
18	aspaewy	822.8648	822.3548	823.3627	412.1853
19	wspesGy	824.8377	824.3341	825.3419	413.1749
20	wrpasGy	835.9100	835.3978	836.4057	418.7067
21	aspeswy	838.8643	838.3497	839.3576	420.1827
22	**arpaswy**	**849.9366**	**849.4135**	**850.4213**	**425.7146**
23	**wspeiGy**	**850.9183**	**850.3862**	**851.3940**	**426.2009**
24	wrpaiGy	861.9906	861.4499	862.4577	431.7328
25	aspeiwy	864.9449	864.4018	865.4096	433.2087
26	wspeeGy	866.8746	866.3447	867.3525	434.1802
27	arpaiwy	876.0172	875.4655	876.4734	438.7406
28	wrpaeGy	877.9469	877.4084	878.4162	439.7120
29	aspeewy	880.9012	880.3603	881.3681	441.1880
30	arpaewy	891.9735	891.4240	892.4319	446.7198
31	wrpesGy	893.9464	893.4033	894.4111	447.7095
32	wspaswy	895.9606	895.3865	896.3943	448.7011
33	arpeswy	907.9730	907.4189	908.4267	454.7173
34	wrpeiGy	920.0270	919.4553	920.4631	460.7355
35	wspaiwy	922.0412	921.4386	922.4464	461.7271
36	arpeiwy	934.0536	933.4710	934.4788	467.7433
37	wrpeeGy	935.9833	935.4138	936.4216	468.7147
38	wspaewy	937.9975	937.3971	938.4049	469.7064
39	arpeewy	950.0099	949.4295	950.4373	475.7226

40	wspeswy	953.9970	953.3920	954.3998	477.7038
41	wrpaswy	965.0693	964.4557	965.4635	483.2357
42	wspeiwy	980.0776	979.4440	980.4519	490.7298
43	wrpaiwy	991.1499	990.5077	991.5156	496.2617
44	wspeewy	996.0339	995.4025	996.4103	498.7091
45	wrpaewy	1007.1062	1006.4662	1007.4741	504.2410
46	wrpeswy	1023.1057	1022.4611	1023.4690	512.2384
47	wrpeiwy	1049.1863	1048.5132	1049.5210	525.2644
48	wrpeewy	1065.1426	1064.4717	1065.4795	533.2437
